# Molecular indices of viral disease development in wild migrating salmon^†^

**DOI:** 10.1093/conphys/cox036

**Published:** 2017-06-27

**Authors:** Kristina M. Miller, Oliver P. Günther, Shaorong Li, Karia H. Kaukinen, Tobi J. Ming

**Affiliations:** 1 Fisheries and Oceans Canada, Pacific Biological Station, 3190 Hammond Bay Road, Nanaimo, British Columbia, Canada V9T 6N7; 2 Günther Analytics, 402-5775 Hampton Place, Vancouver, British Columbia, Canada V6T 2G6

**Keywords:** viral disease, host transcriptome, disease biomarkers, wild populations, aquaculture, salmon

## Abstract

Infectious diseases can impact the physiological performance of individuals, including their mobility, visual acuity, behavior and tolerance and ability to effectively respond to additional stressors. These physiological effects can influence competitiveness, social hierarchy, habitat usage, migratory behavior and risk to predation, and in some circumstances, viability of populations. While there are multiple means of detecting infectious agents (microscopy, culture, molecular assays), the detection of infectious diseases in wild populations in circumstances where mortality is not observable can be difficult. Moreover, if infection-related physiological compromise leaves individuals vulnerable to predation, it may be rare to observe wildlife in a late stage of disease. Diagnostic technologies designed to diagnose cause of death are not always sensitive enough to detect early stages of disease development in live-sampled organisms. Sensitive technologies that can differentiate agent carrier states from active disease states are required to demonstrate impacts of infectious diseases in wild populations. We present the discovery and validation of salmon host transcriptional biomarkers capable of distinguishing fish in an active viral disease state [viral disease development (VDD)] from those carrying a latent viral infection, and viral versus bacterial disease states. Biomarker discovery was conducted through meta-analysis of published and in-house microarray data, and validation performed on independent datasets including disease challenge studies and farmed salmon diagnosed with various viral, bacterial and parasitic diseases. We demonstrate that the VDD biomarker panel is predictive of disease development across RNA-viral species, salmon species and salmon tissues, and can recognize a viral disease state in wild-migrating salmon. Moreover, we show that there is considerable overlap in the biomarkers resolved in our study in salmon with those based on similar human viral influenza research, suggesting a highly conserved suite of host genes associated with viral disease that may be applicable across a broad range of vertebrate taxa.

## Teaser

Impacts of infectious diseases on wildlife populations can be difficult to document when mortality is not observable. We present a technology that utilizes a highly conserved host response to viral disease to differentiate latent viral infections from active disease states and viral from bacterial diseases.

## Introduction

Aquatic fishes are naturally exposed to a wide array of infectious agents that can impact their performance and survival, yet mere exposure to a pathogen does not always result in disease development. Disease development is a manifestation that depends upon host susceptibility, pathogen virulence and environmental conditions (Scott, 1988; Hedrick, 1998; Harvell *et al.*, 2009), and disease ensues when the host sustains sufficient damage to perturb homeostasis (Casadevall and Pirofski, 1999). Compromised immunity in individuals living under stressful environmental conditions or those already responding to pre-existing infections can enhance disease susceptibility (Wedemeyer, 1970, 1996; Harvell *et al.*, 2009).

Highly virulent pathogens can cause acute diseases that affect both healthy and compromised individuals in a population. However, while we know that their impacts can be devastating under high density culture conditions where contact rates between infected and uninfected individuals is high (Anderson and May, 1986), there is some question whether acute diseases, often caused by viral infections, result in the same population-level impacts in wild populations due to reduced transmission potential at low densities. The exception would be fish species that school at high densities, such as herring, for which massive mortality events due to viral hemorrhagic septicemia virus (VHSv) have been observed (Skall *et al.*, 2005). Alternately infectious agents that cause chronic infections, allowing for broader transmission potential in low density populations, and impacting physiological performance rather than directly causing mortality, may, in fact, be more impactful in lower density fish populations (Miller *et al.*, 2014).

Traditional fish health investigations generally start with observed mortality, and utilize diagnostic methods to identify the cause of death of individuals in a late- or final-stages of disease. Histopathology visualizes damage to the tissue to identify the cause of death, to classify the disease, and if infectious, to identify directly or to propose the pathogen(s) that are likely causative. Parasitic and bacterial infections may be observable microscopically, but unless viruses form inclusion bodies, they are not generally visible. However, once a pathogen is suspected, targeted immunohistochemistry or in situ hybridization can be used to verify the presence of a specific agent, including viruses, and to localize the agent within the region of tissue damage. Pathogen culture can also be attempted for suspected viral or bacterial infections, but not all pathogenic species are amenable to culture. All of these approaches are most effective at resolving late-stage disease. Molecular technologies have improved diagnostics for viruses and other pathogens difficult to assay by traditional means (Hungnes *et al.*, 2000; Lafferty *et al.*, 2004), and can provide highly sensitive detections (Mackay *et al.*, 2002). Recently, multi-pathogen monitoring systems for characterized agents have been developed using molecular [microfluidics qPCR (described below); Miller *et al.*, 2016] and serological (VirScan; Lungat and Petrova, 2015) technologies, and metagenomics has been applied to identify novel agents (Mokili *et al.*, 2012).

The challenges to our understanding of the role of infectious diseases in wild fish are numerous. In many environments, like the ocean, mortality events may not be readily observable, as dying fish may sink down the water column or be quickly taken by an array of marine, avian and terrestrial predators. Moreover, infectious diseases that impact physiological performance—e.g. swimming ability, visual acuity, schooling, feeding behavior, ability to maintain ion homeostasis—may considerably enhance risk of predation (Miller *et al.*, 2014). In such cases, where predators are abundant, it may be rare for infectious disease to directly kill fish, and as such, it may be difficult to sample fish at a late stage of disease development. As a result, while laboratory challenge studies may resolve pathogens capable of causing disease and impacting performance of wild fish, actually documenting their impacts in a wild context may require a new generation of tools that are more sensitive to resolving earlier stages of disease development than those traditionally utilized in the fish health diagnostic community.

We recently developed a high throughput molecular microfluidics approach to quantitate dozens of infectious agents (viruses, bacteria, fungal and protozoan parasites) in salmon in 96 samples at once (Miller *et al.*, 2016). This platform has been applied to resolve the spatial and temporal distributions of infectious agents in migratory salmon (Miller *et al.*, 2014), and could be easily modified to conduct strain-typing within a species to identify virulence factors or to aid in the interpretation of specific disease outbreaks, as has been done for human *Streptococcus* strain variants (Dhoubhadel *et al.*, 2014). With our multi-agent quantitative monitoring system, we find that in wild migratory salmon, mixed infections are common, and few fish are agent-free. However, most individuals do not carry multiple infectious agents at appreciable loads (i.e. abundances). While pathogen loads are not a direct indication of disease, for pathogens causing acute diseases, there is greater potential for disease manifestation (i.e. damage) at higher loads (Johnson *et al.*, 2004). In fact, truncation in load distributions, whereby there are fewer individuals carrying high loads of an infective agent than expected under a normal distribution, has been one method used by parasitologists to identify potential linkages between parasites and mortality in field-based studies (Lester, 1984). However, documenting a physiological impact, be it at molecular, protein, cellular or whole organism level, would be a more direct and powerful means to demonstrate disease manifestation.

In the human health arena, molecular diagnostics of the host are increasingly being utilized to identify diseases and to characterize the molecular basis of cellular damage for the development of targeted therapeutants (Ross and Ginsburg, 2003). Because tissue damage is often caused by disruption of molecular pathways, molecular diagnostic tools can be highly sensitive to the detection of early stage disease even before cellular damage or outward symptoms are apparent (Woods *et al.*, 2013; Andres-Terre *et al.*, 2015). For infectious diseases, molecular tools can potentially distinguish between latent infections, where the agent may be detected but due to lack of pathogen activity there is minimal host response, from active, disease causing infections, where higher loads of infectious agents are present and strong differential activation of immunity and cellular processes that ultimately can lead to tissue damage (i.e. disease) occurs. What is less clear, however, is how well such methodologies would work in situations where multiple infectious agents may be present.

In a recent study on human viral influenza, researchers conducted an integrated, multi-cohort analysis over a broad range of microarray-based transcriptome studies to identify host biomarkers predictive of viral influenza as well as those predictive of general viral respiratory disease (Andres-Terre *et al.*, 2015). Uniquely, instead of carefully controlling a myriad of technical variables by choosing only studies conducted on a single array platform, a single tissue and designed in a similar manner, to increase the robustness of the biomarkers resolved, they instead incorporated variation in molecular platforms, tissues profiled, and included a range of study designs—some contrasting viral influenza versus healthy controls, others viral versus bacterial respiratory infections, with separate studies explored in discovery and validation sets. The biomarkers discovered could discriminate, based on host gene activity alone, individuals developing general viral respiratory infections, viral influenza, bacterial respiratory infections and disease free individuals. Not only did the biomarker panel resolved identify the presence of infectious disease before outward symptoms were present, but the panel was effective across saliva and blood samples. This study provides the foundation of the work that we have undertaken to identify biomarkers predictive of viral disease development (VDD) in salmon that can be applied alongside our broad-based microfluidics pathogen monitoring system to differentiate latent viral infections from the presence of viral disease. We are ultimately interested in expanding this technology to identify bacterial disease development and diseases associated with different families of microparasites.

The study undertaken herein identifies a host VDD biomarker panel that can effectively characterize the development of a viral disease state across a range of hosts, tissues and virus species. We started with a published study by Krasnov *et al.* (2011) in which transcriptome analyses were performed on Atlantic salmon from viral challenge studies for infectious anemia virus (ISAv), infectious pancreatic necrosis virus (IPNv), piscine orthoreovirus (PRv) and piscine myocarditis virus (PMCv), and analyses were undertaken to identify early viral response genes (VRG) differentially regulated across multiple viral diseases. While the VRG were not identified using highly advanced statistical methods, they served as a starting point for our multi-cohort data meta-analyses, which incorporated both our own transcriptome challenge studies and published, publicly available studies. In our approach, we focused much of our refinement of this initial set of biomarkers on in house and published microarray challenge studies based on an RNA virus endemic to salmon in British Columbia that was not part of the Krasnov study (infectious hematopoietic necrosis virus; IHNv). Validation of the 45 biomarker VDD panel was performed by applying microfluidics qPCR on samples from three sets of in-house studies independent of the discovery samples and analyses: (i) IHNv challenge studies to assess the classification ability of the panel across multiple salmon species and tissues, (ii) diagnostic samples from a BC Chinook salmon farm outbreak of viral jaundice/anemia to assess classification ability of fish undergoing a novel viral disease across tissues and (iii) diagnostic samples from moribund/recently dead farmed salmon collected as part of a regulatory audit program to discern whether the panel could distinguish (RNA) viral disease from bacterial and parasite-induced diseases. Our final application of the VDD panel was on naturally migrating Sockeye salmon smolts, some of which carried high loads of IHNv, to determine if a viral disease state could be detected in live-sampled wild fish.

## Materials and methods

### VDD biomarker discovery—published multi-cohort microarray datasets

The research to define a suite of host biomarkers consistently associated with VDD in salmon across a range of RNA viral species began with multi-cohort data from publically available microarray datasets. Data from three published microarray studies were used in the initial identification of biomarkers associated with VDD across viral species. In Krasnov *et al.* (2011), the salmon SIQ array was applied to a series of challenge studies on Atlantic salmon based on four different salmon viruses, ISAv, IPNv, PMCv and PRv. This study aimed to resolve consistent VRG differentially regulated in early disease development for all RNA viruses in salmon, very similar to the objective of our research. Our study additionally aimed to determine if a viral disease state could be predicted both in the primary infective tissue and in non-destructive gill tissue, even for infections whereby gill tissue is not a primary target of the virus, and whether we could identify a panel that could additionally differentiate viral from bacterial disease states. The panel of 25 VRG identified by Krasnov and colleagues were utilized in virtually all evaluation and validation steps performed herein based on studies independent of the Krasnov study. However, as most of the published and in house studies explored herein were based on GRASP salmon arrays (16K and 32K; GRASP web.uvic.ca/cbr/grasp; B.F. Koop and W. Davidson), we first mapped the features defined in Krasnov across the SIQ and GRASP arrays for microarray exploration.

The second study considered was by Skjesol *et al.* (2011), which applied the SFA 2.0/immunochip (GPL6154; UKU_trout_1.8K_v1) on head kidney cDNA from Atlantic salmon challenged with two different field isolates of IPNv showing different levels of virulence [NFH-Ar (virulent) versus NFH-El (avirulent)], documenting host response elicited by each at 13 days post injection (five samples each plus four controls). The third study considered was by Purcell *et al.* (2011), which applied the GRASP16k array on anterior kidney cDNA from Rainbow trout challenged with virulent and avirulent strains of IHNv [4 controls, 4 IHNv M-type (virulent strain) and 4 IHNV U-type (avirulent strain)].

Significant gene lists from each of these published challenge studies were combined to form signature CS0301u, representing the union of 532 features which formed the basis of exploratory analyses and refinement of the biomarker panel based on analyses of independent in-house IHNv microarray datasets (described below).

A fourth published study by LeBlanc *et al.* (2010), whereby the cGRASP32K array was applied on head–kidney cDNA from Atlantic salmon at multiple time points post injection challenge with ISAv (81 samples including controls), was not used in the initial analyses, but the consistency in the directional activity of VDD biomarkers eventually identified was compared back to the findings of this study.

### Molecular Genetics Laboratory (MGL) IHNv challenge datasets—refinement of viral disease biomarkers and qRT-PCR validation of VDD panel across species and tissues

In 2005, we conducted a series of IHNv challenges (ip-injection and waterborne) on four salmon species [Atlantic (*Salmo salar*), Sockeye (*Onchorhynchus nerka*), Chum (*O. keta*) and Chinook (*O. tshawytscha*)] carrying different susceptibilities to the IHN virus (listed most to least, respectively). Anterior kidney collected from a portion of the fish from the ip-challenges was used in microarray studies, as described in Miller *et al.* (2007). Herein, we explored the data from the microarray challenge studies to refine the CS0301u signature panel by identifying the features most consistently differentially regulated among salmon species as they developed IHN. We utilized the remaining fish and tissues from ip- and waterborne challenge studies as one of the four datasets applied to the qRT-PCR validation of the VDD panel.

Pacific salmon used in challenge studies were obtained from DFO hatcheries and moved to the fish holding facilities at the Pacific Biological Station. Atlantic salmon were obtained from a commercial freshwater production site. All experimental challenges were conducted with post-smolts, after gradually switching from fresh to salt water an acclimation period of several weeks was allowed prior to challenge. Experimental groups of fish were challenged with IHN virus (strain 93-057; genogroup U) by intraperitoneal injection [0.1 ml with IHN virus containing 1.4−2.8 × 10^4^ plaque forming units [pfu] (slight variation among species)] and by waterborne exposure to the virus (8.0 × 10^3^–1.2 × 10^4^ pfu/ml) for 1 h. Control fish were injected with 0.1 ml of sterile Hanks balanced salt solution.

Within each challenge study, five salmon were sampled on alternate day's post-challenge, and anterior kidney, liver, spleen and gill tissues were removed and flash frozen at −80°C until extraction. For each species, five control samples were also collected from uninfected fish at time point zero. Fish handling and microarray protocols followed Miller *et al.* (2007), and herein we only present the variances from this study (based on an Atlantic salmon ip-challenge). All microarray studies were performed using the salmon GRASP16K microarray. A pooled reference design was utilized to calculate relative gene expression ratios across samples. For all but Atlantic salmon, the standard reference was constructed by pooling the total RNA extracted from four tissues (gill, spleen, head kidney and liver) collected from Sockeye salmon during the injection challenge. In Atlantic salmon, a pooled reference that combined total RNA from all kidneys collected in an Atlantic salmon waterborne challenge experiment was used.

Samples available from challenge studies are shown in Table 1A, which also depicts (in parentheses) the portion of samples analyzed for biomarker refinement from microarray studies. In each microarray study, a time-series of samples post-challenge (5 per collection date) was compared to mock-challenged fish collected on Day 0.

**Table 1:** Experimental study designs for biomarker validation studies, by species and tissues sampled. (A) Total samples analyzed from IHNv challenges, with the subset analyzed in microarray analyses to facilitate biomarker discovery-refinement shown in parentheses. (B) Chinook salmon farm samples collected during a jaundice/anemia outbreak. Disease status was determined by a veterinarian at the farm site and confirmed via histopathology. Healthy controls were a combination of healthy fish from the same farm and fish from an adjacent farm with no jaundice. (C) Farm audit samples collected between 2011 and 2013 by quarter. Audit samples include moribund/recently dead samples from randomly selected farms throughout British Columbia. Mixed-tissue RNA samples for each individual were analyzed with the VDD biomarkers. (D) Gill samples from Sockeye salmon smolt outmigrants. Collections occurred over 3 years, 2007, 2011 and 2012 at the Fraser River Chilko Lake smolt fence. In 2012, smolts were acoustically tagged and tracked (Jeffries *et al.* 2014). 2007 was a year of very poor smolt survival

A.					
Tissue	Challenge	Sockeye	Atlantic	Chum	Total
Head kidney	Injected	45 (45)	40 (25)	45 (20)	
	Waterborne	50	116	45	
Gill	Injected	45	5		
	Waterborne	45	115	43	
Liver		45			
	Injected	45			
Spleen		45			
	Injected	45			
Grand total		275	276	133	**684**

B.		
Tissue	Total	Jaundice/anemia
Gill	36	16
Head kidney	35	16
Liver	36	16
Spleen	31	13
Heart	36	16
Grand total	**174**	

C.				
Year	Quarter	Atlantic	Pacific	Total
2011		**116**	**32**	
	1			
	2	42	14	
	3	34	6	
	4	40	12	
2012		**46**	**10**	
	1	45	9	
	2	1	1	
	3			
	4			
2013		**78**	**26**	
	1	1	1	
	2		1	
	3	39	16	
	4	38	8	
Grand total		240	68	308

D.	
Year	*N*
2007	14
2011	18
2012	181

### Jaundice syndrome dataset—qRT-PCR validation of VDD panel across tissues on a natural viral disease outbreak

Jaundice syndrome is a disease impacting farmed Chinook salmon in BC that holds a striking resemblance to a disease in farmed Coho salmon in Chile (Godoy *et al.*, 2016) and farmed Rainbow trout in Norway (Olsen *et al.*, 2015). All are suspected to be viral-induced, with the PRv being the one virus common to outbreaks in all three species/countries; there has been no research to date to determine the nature of the relationship between PRv and these outbreaks. Anterior kidney, liver, heart, spleen and gill tissues were collected during a farm outbreak of jaundice syndrome, clinically characterized by jaundice and anemia, with collections including both sick and dying fish and healthy controls (Table 1B). Disease classification was determined through veterinary diagnostics comprised of clinical and histopathology data.

### Farm audit samples—qRT-PCR validation of VDD panel across viral and bacterial diseases

Samples of dying farmed salmon were made available from the Fisheries and Ocean Canada regulatory farm audit program. These samples are collected to monitor background losses in production populations, to detect ongoing or recent health events within the industry, and to ensure reporting compliance with OIE (World Organization of Animal Health) listed diseases. Farm audit samples are collected on randomized BC farms, with one to six fresh silver (recently dead) fish sampled per farm audit in 2011–13. At the time of collection, clinical and environmental data are noted, and tissue samples are taken for histopathology, bacterial and viral culture and molecular analysis. Veterinary diagnostics were conducted on these samples prior to our application of the VDD, and were based largely on histopathology and clinical data. Our team had already conducted quantitative molecular analyses of 45 infectious agents known or suspected to cause disease in salmon on cDNA/DNA from combined tissues (heart, liver, head and anterior kidney, gill, pyloric caeca, spleen), so the backdrop of known infectious agents was determined for each sample. The VDD biomarkers were applied on this same cDNA from 240 farmed Atlantic salmon and 68 farmed Chinook salmon collected from 2011 to 2013 (Table 1C). We utilized these data to assess the ability of the VDD to discriminate fish experiencing viral- versus bacterial- or parasite-induced diseases based on tissue pools.

### Application to wild salmon

The ultimate goal of the VDD biomarker development was to develop a tool that could not only identify the distribution of infectious agents in wild migratory salmon, but could also discriminate between latent viral carrier and disease states using a non-destructively sampled tissue. Throughout our larger research program we have utilized gill tissue (tips of 1–2 gill filaments) to biopsy fish with minimal impact on performance (reviewed in Cooke *et al.*, 2005). Our final VDD validation study was based on Sockeye salmon smolts leaving their freshwater natal rearing lake (Chilko Lake in the Fraser River, BC). In 2012, we conducted an acoustic tracking study and assessed linkages between 18 infectious agents and 50 stress and immune-related genes and migratory fate. We showed that fish with high loads of IHNv and those containing a correlated antiviral type signature (including up-regulation of MX and STAT1, the two genes overlapping with the VDD biomarkers) generally disappeared within the first 80 km of downstream migration (Jeffries *et al.*, 2014), likely due to enhanced risk of predation by resident Bull trout (Furey, 2016). We applied the validated VDD biomarkers on the 213 smolts from the Jeffries study collected at the Chilko smolt fence in 2007, 2011 and 2012 to determine if there was evidence of viral disease in these fish (Table 1D).

## Statistical approach

### Meta-analysis for VDD discovery

**Figure 1:**
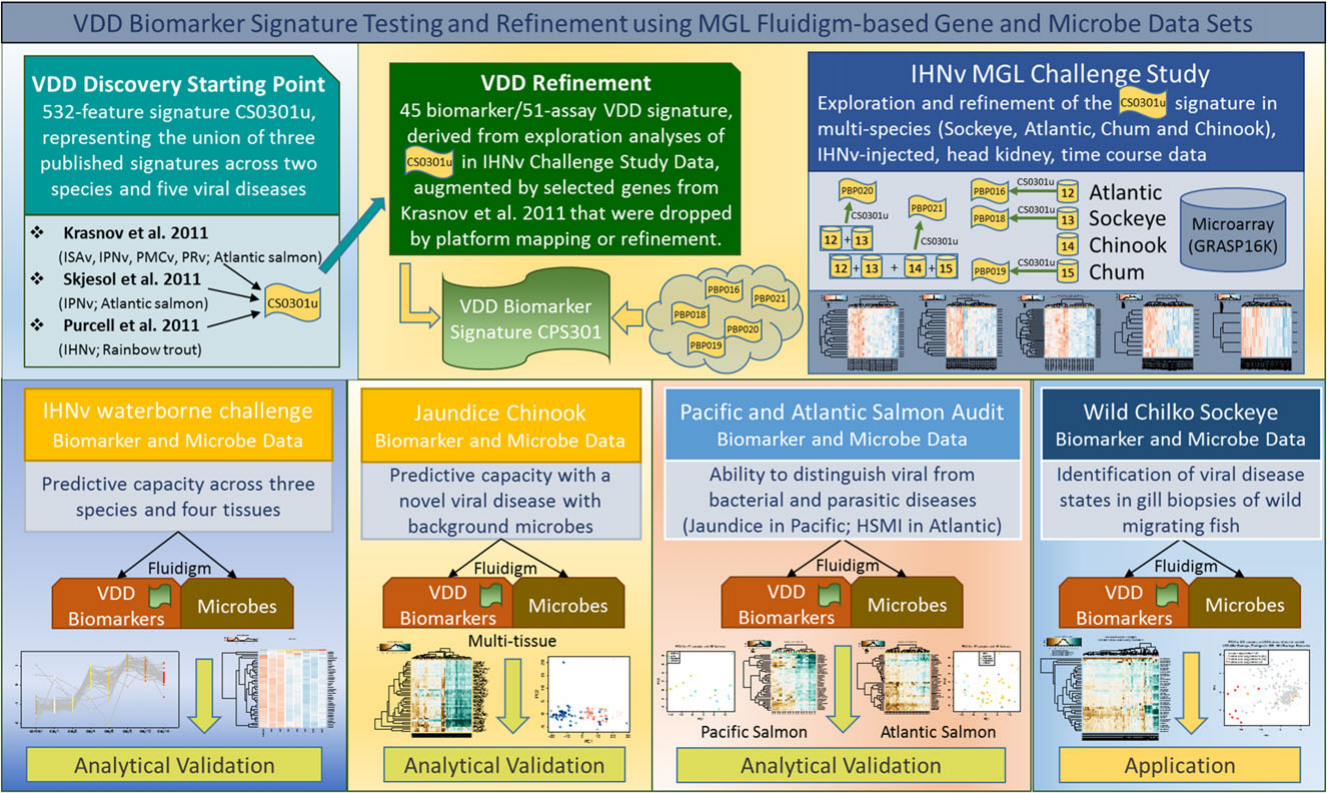
Schematic of viral disease development (VDD) discovery, refinement, validation and application datasets. The VDD discovery dataset was identified from published microarray viral challenge studies that included five RNA virus species. In house (MGL) IHNv challenge microarray studies across four salmon species were used to refine the VDD panel. Analytical validations of the qRT-PCR assays developed to 45 biomarkers within the VDD panel was performed using independent in-house studies that tested discrimination abilities of the proposed VDD between latent and disease-associated viral infections across tissues, salmon and viral species, as well as differentiation of fish undergoing viral, bacterial, and parasitic diseases. The VDD panel was then applied to wild migrating Sockeye salmon smolts to discern whether wild fish infected with IHNv could be identified in a VDD state.

Analyses for the development of the VDD biomarkers was broken up into four segments (see schematic in Fig. 1). The first three analysis segments utilized microarray datasets. The first segment simply combined the significant gene lists from the three published studies (into signature CS0301u), after first mapping the genes for each onto the GRASP microarrays upon which our own studies were based. The second analysis segment involved refining the CS0301u signature set by meta-analysis of the multi-species microarray datasets from our own IHNv challenges. In addition to validating signature CS0301u on Atlantic, Sockeye and Chum salmon challenge data (resulting in feature panels PB0P16-PBP019), this signature was also validated in combined Atlantic and Sockeye (PBP020), and combined Atlantic, Sockeye, Chum and Chinook salmon challenge datasets (PBP021) (Table S1). This multi-species, multi-signature approach focused on identifying robust biomarkers across species and viral pathogens. In each exploration analysis, genes with strong discrimination capabilities were identified using unsupervised clustering approaches. Unsupervised methods are exploratory in nature, but they can be applied to a subset of validation data based on a specific signature to identify a smaller set of genes that consistently separate groups of interest. We utilized the gene shaving algorithm which provides an automatic feature selection by applying principle component analysis (PCA) iteratively to ‘shave off’ genes and create a sequence of clusters with different sizes (Hastie *et al.*, 2000). Final cluster selection is based on a comparison of specific cluster-variance measures with similarly derived measures for randomized data. This method offers the most ‘coherent’ subset of genes across a sequence of cluster candidates.

The third analysis segment involved exploration of the overlap between feature panels resolved by gene shaving (described above) and the consistency of their directional response both within the in-house and published microarray studies. These analyses yielded a feature panel of 38 biomarkers, coined CPS301, that were up-regulated across a range of RNA-viral challenge studies and salmon species based on the primary infective tissues for each (Table S2). As there were a number of potentially important genes resolved by Krasnov *et al.* (2011) that either did not map to the GRASP16K array or were removed due to quality issues, we added 15 additional biomarkers to our proposed VDD panel. This panel included some paralogs to the same genes (from Krasnov).

### Development of TaqMan assays for universal deployment across multiple salmon species

In order to develop TaqMan assays specific to the gene paralog of interest, all proposed VDD biomarkers were mapped onto the Atlantic salmon genome and gene paralogs identified. Where available, sequence alignments were generated to include gene paralogs from Atlantic and Pacific salmon species, and assays were designed to match only one paralog across all species. In general, we designed and tested 2–3 assays per targeted gene paralog. Assay efficiencies were determined on the Fluidigm BioMark HD platform using serial dilutions of mixed tissues derived from each species. Assays with efficiencies between 0.9 and 1.1 were considered optimal. Occasionally, assays did not work across all species, and alternate assays had to be used in some species. In some instances, none of the assays worked for certain species. Some of the proposed VDD genes did not have sufficient sequence data across species to design effective assays; these were dropped from our analysis. In total, 51 TaqMan assays to 45 biomarkers (some to paralogs of the same gene) were developed for validation across three independent datasets (Table 2).

**Table 2:** TaqMan assays applied in validation studies, including host VDD biomarkers, 3 host housekeeping genes and 12 infectious agents, performed on host cDNA. For the full list of assays used in the Chinook salmon jaundice study and audit studies, see Miller *et al.* (2016)

Description	Assay name	Gene ID	Biomarker origin	F Sequence	R sequence	Probe Sequence	EST	Assay efficiencies					
VDD biomarkers								Atlantic	Sockeye	Chum	Chinook	Coho	Pink
Barrier to autointegration factor	BAF_MGL_4	BANF1	Krasnov	CCAACTGAACCATGTCTGGAAA	GTCCCGGTGCTTTTGAGAAG	AAGGAAGGACCCCCC	BT049316	0.83					
Unknown protein [Siniperca chuatsi]	CA038063_MGL_1		CPS301	TGTCCCTCTTCAAGACCTCGTT	AACATGTCTTCATTGTTGGTACAAAAG	CAGAAGTGATGAAAGCAG	CA038063	0.86			0.91	1.06	0.88
Mitochondrial ribosomal protein (VAR1)	CA054694_MGL_1	VAR1	CPS301	CCACCTGAGGTACTGAAGATAAGACA	TTAAGTCCTCCTTCCTCATCTGGTA	TCTACCAGGCCTTAAAG	CA054694	0.81	0.83		0.93	0.91	0.85
CD68 molecule	CD68_MGL_3	CD68	CPS301	GATGATGAGGATAAGGAGGACAATC	GGGACTTCGGCACATCTGA	CCACAGCAATGGC	CA048910	0.75	0.89	0.87	0.91	0.93	0.91
CD9 protein	CD9_MGL_2	CD9	CPS301, Krasnov	CTTGATCTGTTTCATGAGGATGCT	ACCTCCTCCTGTTGCTCCTAGA	CAGCACACCAGGGC	CA064247	0.83	0.9	1.02	0.9	0.93	0.83
Similar to interferon-inducible protein Gig2 (CD9)	GIG2-1_MGB1	CD9 (GIG2-L)	CPS301, Krasnov	GAAAAGAGTACTAAAAATCAGGGTGGAT	GGGTGGTTCTGCCTGTCTATG	TCGGCAGGGTTAAGG	CA054168		1			0.89	
	GIG2-1_MGB2	CD9 (GIG2-L)	CPS301, Krasnov	ATCAAAGTCATCGAGGTCATGAAG	GACTCCACTCTGAAGATGATCATACTG	TTACCGAAGAGAACTTATC	CA054168			0.86	0.89		
	GIG2-1_MGB3	CD9 (GIG2-L)	CPS301, Krasnov	AACACTATGCAGTGGAACTGATGAA	GACCATGAGGTGATGCTGGAT	TCTGCATTCAGTGGGAG	CA054168	0.91					
ATP-dependent RNA helicase	DEXH_MGL_1	DDX58	Krasnov-not 16K	CCATAAGGAGGGTGTCTACAATAAGAT	CTCTCCCCCTTCAGCTTCTGT	TGGCGCGCTACGTG	FN396359	0.78	0.97	1.19	0.86	0.96	0.83
	DEXH_MGL_3	DDX58	Krasnov-not 16K	TGGAGAAGAAGGGTGTGACAGA	CGCAGGTGGAGAGCACACT	AGGAACAGACTGCTGGC	FN396359	0.9		0.88			
RNA helicase—RIG-I	RIG1_MGLSYBR_1	DDX58 (RIG1)	Krasnov	GACGGTCAGCAGGGTGTACT	CCCGTGTCCTAACGAACAGT	TGTCCAATTTAGGATTCTCCTTCTGCCC	DY714827	0.83	0.83	0.86	0.84	1.01	0.9
Slime mold cyclic AMP receptor	DICTY-CAR_MGL_2	DICTY	CPS301	TCAACTTTGACAGTGGTCAGATAGC	TCCTTTTTTCCTCCTTATGATTGG	TGAGGTAGAAGTTGCCTTT	CB494001	0.91	0.97	0.92	1	1.12	0.95
Galectin-3-binding protein precursor	GAL3_MGL_2	LGALS3BP	CPS301	TTGTAGCGCCTGTTGTAATCATATC	TACACTGCTGAGGCCATGGA	CTTGGCGTGGTGGC	CB515011	0.95	1	1.03	1.11	1.03	0.89
Guanine nucleotide-binding protein-like 3	GNL3_MGL_1	GNL3	CPS301	GCCCAGTCTAACCCAAAAGCT	GGGTCCTGACGGCCTCTAG	CCATGGCGCTGAGG	CB499134	0.87	0.84	0.84	0.86	1.01	0.89
Similar to KIAA1593	KIAA_MGL_1	HERC4	Krasnov-not 16K	GATCGCTACCTTCATCTGAATCTTG	CTGTTCTTGACGGGCTGTGA	CATGCCCAGGATGG	EG841846		0.76	1.12	0.87	0.79	0.87
Probable E3 ubiquitin-protein ligase HERC6	HERC6_1	HERC6	CPS301	AGGGACAACTTGGTAGACAGAAGAA	TGACGCACACACAGCTACAGAGT	CAGTGGTCTCTGTGGCT	CA060884	0.87	0.86	1.07	0.85		
IFN-induced protein	IFI_MGB2		Krasnov	GCTAGTGCTCTTGAGTATCTCCACAA	TCACCAGTAACTCTGTATCATCCTGTCT	AGCTGAAAGCACTTGAG	NM_001 124 333	1.01	0.93	0.96	0.98	0.91	0.85
Interferon induced with helicase C domain 1—IC	IFI-1C_MGL_2	IFIH1 (MDA5)	Krasnov	TCCCCAGAGCAGACTGGTTT	AGAGCCCGTCCAAAGTGAAGT	TTGCAGCTTCTACAACTG	GE823089	0.79	0.88	0.9	0.9	0.99	0.89
Interferon-induced 35 kDa protein homolog	IFI35_MGL_2	IFI35	CPS301	CAACCAAGCCAGGGATGTAGA	GCTCTCTGGATCTCCCTCTTCA	AAAGGAAGAAGATAGCCGCC	CA064047	0.78	0.79		0.92	0.85	
IFN-induced protein 44-1	IFI44A_MGL_2	IFI44	Krasnov-not 16K	CGGAGTCCAGAGCAGCCTACT	TCCAGTGGTCTCCCCATCTC	CGCTGGTCCTGTGTGA	GS365948	0.8	0.71		0.73	0.76	1.18
	IFI44C_MGL_3	IFI44	Krasnov	GGCAAACCGCTGCCAAT	CCCTGTGGCCTCCTCCAT	TTTTGTGTGACACGATGGG	EV384577	0.85	0.81	0.84	0.88	0.87	0.8
Interferon-induced protein with tetratricopeptide repeats 5	IFIT5_MGL_2	IFIT5	CPS301, Krasnov	CCGTCAATGAGTCCCTACACATT	CACAGGCCAATTTGGTGATG	CTGTCTCCAAACTCCCA	CA051350	0.97	0.93	0.9	0.98	1.03	0.95
Interferon regulatory factor 7	IRF7_MGL_2	IRF7	CPS301	ACACCCTGAACCCAGGAAGA	AAAGCACATGTGGATGGTATAGTCA	CAAAATGAAATGGTACAACTG	CN442559	0.89	0.96	1.07	0.97	1.05	0.86
Interferon-induced GTP-binding protein Mx	MX	MX1	In house	AGATGATGCTGCACCTCAAGTC	CTGCAGCTGGGAAGCAAAC	ATTCCCATGGTGATCCGCTACCTGG	CB516446	0.65	0.78	0.9	0.97	N/A	N/A
Zinc finger NFX1-type	NFX_MGL_2	NFX1type	Krasnov	CCACTTGCCAGAGCATGGT	CGTAACTGCCCAGAGTGCAAT	TGCTCCACCGATCG	FQ635861	0.83	0.87	0.73	0.84	0.81	0.84
PLAC8-like protein 1	PLAC8L1_MGL_1	PLAC8L1	CPS301	GAGAACGCTACGGCATCCA	CCATCTGGCACCAGGTACAGA	CATTGGTGTGTTGCTGC	CA047116	0.89	0.87	0.84	0.88	0.98	0.85
Urokinase plasminogen activator surface receptor precursor	PLAUR_MGL_3	PLAUR	CPS301	CAGTCTCCACTATCTACCTGTTGTGTGT	TGTGACGCCCCAAGGAA	AGCCCCTTTCACTGGA	CA057830	0.82	0.97	0.82	0.96	1.02	0.89
Proteasome subunit beta type-8 precursor	PSMB8_1	PSMB8	CPS301	CATGTCTGGTAGTGCTGCTGACT	TCTGCTTGTTCCTCAGTTTGTACAG	CAGTACTGGGAGAGACT	CA061622	0.81	0.84	0.85	0.86	0.93	0.86
Proteasome subunit beta type-9 precursor	PSMB9A_MGL_2	PSMB9	CPS301	GTTGCCCAGGATGCATTTCT	CCATGAGTCGAGATGGTTCGA	ATAGTGACAAGGTAGGCCAC	CA064302	0.87	0.85	0.91	0.99	0.93	0.91
Retinoic acid-inducible gene-1	RAD_MGL_2	RAD1	Krasnov	GGTGATGAGGAGGAGGGTGAA	CAAACTGCTCGGTGTACTGGAA	CCATGACGACTATCTC	FN178459	0.88					
RING finger protein 213	RNF213_MGL_5	RNF213	CPS301	GTAATATGAGTGACGTGAAAGTG	TCGGTCGATCTCTGTGT	TTTGTGGACCTGGCCTCCATCTC	CA053171	0.93	0.92	0.89	0.93	1.05	1.01
UNKNOWN	E3RNF213_2	RNF213	CPS301	CTCCAGATTCTCCAGCAGACATT	GTACTCTTGATCCTTTGGGAAGCT	TTCTCAGACCACAACCAT	CA059288	0.81	0.82	0.9	0.9	0.96	0.83
Radical S-adenosyl methionine domain-containing protein 2	RSAD_MGB2	RSAD2 (viperin)	CPS301, Krasnov	GGGAAATTAGTCCAATACTGCAAAC	GCCATTGCTGACAATACTGACACT	CGACCTCCAGCTCC	CA038316	0.89	0.79	0.89	0.85	1.02	0.8
Receptor-transporting protein 3	RTP3_MGL_1	RTP3	Krasnov	TTCCATTAAGGCAGACAGTGTGA	TCCAAATGCCCCACTGATGT	ATCAGGCTGGCATCAG	EG825775	0.79					
Sacsin	SAC_MGB1	SACS	Krasnov	TCAGTCAGGCCCAGTGTGATC	GGCCCTGCCTCCTGTGT	AGCTGCTGCTCACAAC	EG906096		0.88		0.96	0.97	0.97
	SAC_MGB2	SACS	Krasnov	GTACATCAGGCCGTGGAGAAG	GGAGATGGAGCTGTCTTTGTAATAATG	TGTCTTCTGTACTCTGCTGCCACC	EG906096	0.87					
Tyrosine-protein kinase FRK	SRK2_MGB3	FRK	CPS301, Krasnov	CCAACGAGAAGTTCACCATCAA	TCATGATCTCATACAGCAAGATTCC	TGTGACGTGTGGTCCT	CB492720	0.92	0.82	0.96	1.17	0.95	1
Signal transducer and activator of transcription 1-alpha/beta	STAT1	STAT1	CPS301	TGTCACCGTCTCAGACAGATCTG	TGTTGGTCTCTGTAAGGCAACGT	AGTTGCTGAAAACCGG	CA050950	N/A	1.03	0.82	0.76	N/A	N/A
Fish virus induced TRIM-1	TRIM1_MGB1	TRIM1	Krasnov-not 16K	CATGATGTCTGGTGTTGATGTATATTG	GAGACAGAGAACCAACTGAGAAAACATA	TTGTCATTCAGAACCATTG	AM887808	0.99	0.94	0.94	0.99	0.96	0.96
52 kDa Ro protein-2 − 52Ro	52RO_MGL_3	TRIM21	Krasnov	TGCACTATTGCCCAGTAACCAT	TGCAAGAGGAGATGCCAACA	AGTAGGATTCACAGAGAGTT	CX141267		0.85	1.04	0.99	1.11	0.91
MHC class I antigen (*Salmo salar*)	UBA_MGL_CA050178_1	MHC1uba	CPS301	GATCTACTCCGTTCCAGCCATT	TATGGATCTGTGTTTACAGTGTGTGTGT	TTATGATCTGTCCTCCCC	CA050178	0.91					
	UBA_MGL_CA050178_4	MHC1uba	CPS301	CAGTAAGATATGTTCTAAACAGCAAAGGA	CAGCATCTTTCATACAGATCATCAAA	TGTATATGGGTTTAAGAAGAAG	CA050178		0.88			0.95	0.82
Ubiqitin-like protein-1, Peroxisomal membrane protein 2	UBL1_MGL_2	PXMP2	CPS301, Krasnov	GGCCTGCATTCAGGATCTAA	TACAGTCTCACCAGGCACCA	AGTGATGGTGCTGATTACGGAGCC	CB499972	0.61	0.95	0.91	0.94	0.63	0.89
Ubl carboxyl-terminal hydrolase 18	USP18_MGL_2	USP18	CPS301	TTCCAGCTAACCTGCCGTACA	CAGTACAGTTTGTGTGCAGTCATAGTG	TATGCTGTGTAGTGTCCAAA	CA056962	0.96	0.88	0.82	0.95	0.74	0.93
VHSV-inducible protein-1	VHSVIP1_MGL_3	VIG1	Krasnov-not 16K	TGGCTTCCCACATTGCAA	CCTCCTCCCCCCTGCAT	AGATGGAGACAGGAATG	AF483546		0.63	0.83	0.85	0.87	
VHSV-inducible protein-4	VHSVIP4_MGL_3	VIG4	Krasnov	GCTCTCGTAAAGCCCCACATC	GGGCGACTGCTCTCTGATCT	AAACTGCACGTCGCGC	GO053979	0.93		0.66	0.6	0.87	
VHSV-induced protein-10 mRNA	VHSV-P10_MGL_2	VIG10	CPS301	GCAAACTGAGAAAACCATCAAGAA	CCGTCAGCTCCCTCTGCAT	TGTGGAGAAGTTGCAGGC	CA040505	0.79	0.87	0.83	0.93	0.87	0.91
Very large inducible GTPase 1-1	VLIG1-1_MGL_2	GVINP1	Krasnov	CAACAGAGGCCTCAGCAATG	TCTGGCCTCTCCCTGAACTG	ATCACTCCTGGACATGAA	EG841455	0.96		0.92	1.03	1.07	
XIAP-associated factor-1	XAF1_MGL_1	XAF1	Krasnov-not 16K	CGTAGCTACTGGTTTTGGAATCAG	CAGGTTGTGTCCTCTTCCTTGTC	ATTGACAGGTTTCCGCG	BT049703	0.86					
	XAF1_MGL_2	XAF1	Krasnov-not 16K	TGCGGACGCTACATCACTCT	TTGAGGTCAGGGCAGATCTGA	ACCAGCCAGAGCAT	BT049703				0.9	0.89	
PR domain zinc finger protein 9	ZFP9_MGL_2	ZFP9	Krasnov	CGGCTATAAAAAGCCAACTCACA	ACAGTGGTTATAGAGGGTGCAACA	TTATCCCTGAGGTGCTGAC	DQ246664			0.86	1.08	1.07	0.93

Description	Assay name	Gene ID	Biomarker origin	F sequence	R sequence	Probe sequence	EST
Housekeeping genes							
S100 calcium binding protein	78d16.1	78d16.1	Microarray studies	GTCAAGACTGGAGGCTCAGAG	GATCAAGCCCCAGAAGTGTTTG	AAGGTGATTCCCTCGCCGTCCGA	CA056739
Coiled-coil domain-containing protein 84	Coil-P84	Coil-P84	Microarray studies	GCTCATTTGAGGAGAAGGAGGATG	CTGGCGATGCTGTTCCTGAG	TTATCAAGCAGCAAGCC	CA053789
39S ribosomal protein L40, mitochondrial precursor	MrpL40	MrpL40	Microarray studies	CCCAGTATGAGGCACCTGAAGG	GTTAATGCTGCCACCCTCTCAC	ACAACAACATCACCA	CK991258
Infectious agents							
*Candidatus Branchiomonas cysticola*	Bacterium	c_b_cys	Infectious agent	AATACATCGGAACGTGTCTAGTG	GCCATCAGCCGCTCATGTG	CTCGGTCCCAGGCTTTCCTCTCCCA	JQ723599
*Flavobacterium psychrophilum*	Bacterium	fl_psy	Infectious agent	GATCCTTATTCTCACAGTACCGTCAA	TGTAAACTGCTTTTGCACAGGAA	AAACACTCGGTCGTGACC	
*Piscichlamydia salmonis*	Bacterium	pch_sal	Infectious agent	TCACCCCCAGGCTGCTT	GAATTCCATTTCCCCCTCTTG	CAAAACTGCTAGACTAGAGT	EU326495
*Renibacterium salmoninarum*	Bacterium	re_sal	Infectious agent	CAACAGGGTGGTTATTCTGCTTTC	CTATAAGAGCCACCAGCTGCAA	CTCCAGCGCCGCAGGAGGAC	AF123890
*Rickettsia-like organism*	Bacterium	rlo	Infectious agent	GGCTCAACCCAAGAACTGCTT	GTGCAACAGCGTCAGTGACT	CCCAGATAACCGCCTTCGCCTCCG	EU555284
*Moritella viscosa*	Bacterium	mo_vis^a^	Infectious agent	CGTTGCGAATGCAGAGGT	AGGCATTGCTTGCTGGTTA	TGCAGGCAAGCCAACTTCGACA	EU332345
*Tenacibaculum maritimum*	Bacterium	te_mar^a^	Infectious agent	TGCCTTCTACAGAGGGATAGCC	CTATCGTTGCCATGGTAAGCCG	CACTTTGGAATGGCATCG	NBRC15946T
*Yersinia ruckeri*	Bacterium	ye_ruc^a^	Infectious agent	TCCAGCACCAAATACGAAGG	ACATGGCAGAACGCAGAT	AAGGCGGTTACTTCCCGGTTCCC	AY333067
*Ceratomyxa shasta*	Parasite	ce_sha	Infectious agent	CCAGCTTGAGATTAGCTCGGTAA	CCCCGGAACCCGAAAG	CGAGCCAAGTTGGTCTCTCCGTGAAAAC	AF001579
*Cryptobia salmositica*	Parasite	cr_sal	Infectious agent	TCAGTGCCTTTCAGGACATC	GAGGCATCCACTCCAATAGAC	AGGAGGACATGGCAGCCTTTGTAT	
*Dermocystidium salmonis*	Parasite	de_sal	Infectious agent	CAGCCAATCCTTTCGCTTCT	GACGGACGCACACCACAGT	AAGCGGCGTGTGCC	U21337
*Ichthyophonus hoferi*	Parasite	ic_hof	Infectious agent	GTCTGTACTGGTACGGCAGTTTC	TCCCGAACTCAGTAGACACTCAA	TAAGAGCACCCACTGCCTTCGAGAAGA	AF467793
*Ichthyophthirius multifiliis*	Parasite	ic_mul	Infectious agent	AAATGGGCATACGTTTGCAAA	AACCTGCCTGAAACACTCTAATTTTT	ACTCGGCCTTCACTGGTTCGACTTGG	IMU17354
*Loma salmonae*	Parasite	lo_sal	Infectious agent	GGAGTCGCAGCGAAGATAGC	CTTTTCCTCCCTTTACTCATATGCTT	TGCCTGAAATCACGAGAGTGAGACTACCC	HM626243
*Myxobolus arcticus*	Parasite	my_arc	Infectious agent	TGGTAGATACTGAATATCCGGGTTT	AACTGCGCGGTCAAAGTTG	CGTTGATTGTGAGGTTGG	HQ113227
*Parvicapsula kabatai*	Parasite	pa_kab	Infectious agent	CGACCATCTGCACGGTACTG	ACACCACAACTCTGCCTTCCA	CTTCGGGTAGGTCCGG	DQ515821
*Parvicapsula minibicornis*	Parasite	pa_min	Infectious agent	AATAGTTGTTTGTCGTGCACTCTGT	CCGATAGGCTATCCAGTACCTAGTAAG	TGTCCACCTAGTAAGGC	AF201375
*Parvicapsula pseudobranchicola*	Parasite	pa_pse	Infectious agent	CAGCTCCAGTAGTGTATTTCA	TTGAGCACTCTGCTTTATTCAA	CGTATTGCTGTCTTTGACATGCAGT	AY308481
*Sphaerothecum destructuens*	Parasite	sp_des	Infectious agent	GGGTATCCTTCCTCTCGAAATTG	CCCAAACTCGACGCACACT	CGTGTGCGCTTAAT	AY267346
*Tetracapsuloides bryosalmonae*	Parasite	te_bry	Infectious agent	GCGAGATTTGTTGCATTTAAAAAG	GCACATGCAGTGTCCAATCG	CAAAATTGTGGAACCGTCCGACTACGA	AF190669
Infectious hematopoietic necrosis virus	Virus	ihnv	Infectious agent	AGAGCCAAGGCACTGTGCG	TTCTTTGCGGCTTGGTTGA	TGAGACTGAGCGGGACA	NC_001 652
Pacific salmon parvovirus	Virus	pspv	Infectious agent	CCCTCAGGCTCCGATTTTTAT	CGAAGACAACATGGAGGTGACA	CAATTGGAGGCAACTGTA	
Piscine orthoreovirus	Virus	prv	Infectious agent	TGCTAACACTCCAGGAGTCATTG	TGAATCCGCTGCAGATGAGTA	CGCCGGTAGCTCT	
Viral encephalopathy and retinopathy virus	Virus	ver	Infectious agent	TTCCAGCGATACGCTGTTGA	CACCGCCCGTGTTTGC	AAATTCAGCCAATGTGCCCC	AJ245641
Erythrocytic necrosis virus	Virus	env	Infectious agent	CGTAGGGCCCCAATAGTTTCT	GGAGGAAATGCAGACAAGATTTG	TCTTGCCGTTATTTCCAGCACCCG	
Viral hemorrhagic septicemia virus	Virus	vhsv	Infectious agent	AAACTCGCAGGATGTGTGCGTCC	TCTGCGATCTCAGTCAGGATGAA	TAGAGGGCCTTGGTGATCTTCTG	Z93412

^a^Farm audits.

### Application of VDD biomarker TaqMan assays to validate their predictive capacity

TaqMan assays to 45 VDD biomarkers (four with multiple assays required across species) were applied on the Fluidigm BioMark^TM^ HD microfluidics qPCR platform along with TaqMan assays to 23 infectious agents previously detected in a subset of the samples to be tested using the BioMark salmon infectious agent monitoring system outlined in Miller *et al.* (2016). Quantitative infectious agent monitoring for most studies included 5 bacteria, 6 viruses and 12 parasites known or suspected to cause disease in salmon (Table 2). For the audit samples, 50 assays to 49 infectious agents were applied, including all agents outlined in Miller *et al.* (2016) plus three additional pathogenic bacteria known to cause disease on salmon farms: *Moritella viscosa*, *Tenacibaculum maritimum* and *Yersinia ruckeri* (assays in Table 2). These panels were applied to the (i) multi-species IHNv challenge trials to assess performance across species and tissues; (ii) jaundice/anemia Chinook salmon farm outbreak, including multiple tissues to validate performance of the VDD panel on a novel disease hypothesized to be virally induced; (iii) farm audit samples to assess the ability of the VDD panel to differentiate viral versus bacterial or parasitic diseases; and (iv) wild migrating sockeye salmon smolts to discern whether the VDD panel could identify the presence of a viral disease state associated with IHNv infection from non-destructive gill biopsy samples of wild salmon (Jeffries *et al.*, 2014) (experimental designs outlined in Table 1; schematic in Fig. 1).

### Quantitative PCR on the Fluidigm BioMark^TM^ HD platform

Methods for application of TaqMan assays to both host genes and infectious agents have been previously described in Miller *et al.* (2014) and Jeffries *et al.* (2014). Briefly, nucleic acid extractions were performed on homogenizations using Tri-reagent^TM^ using the Magmax™-96 for Microarrays RNA kit (Ambion Inc, Austin, TX, USA) with a Biomek NXPTM (Beckman-Coulter, Mississauga, ON, Canada) automated liquid-handling instrument. RNA was quantitated and normalized to 62.5 ng/μl with a Biomek NXP (Beckman-Coulter, Mississauga, Ontario, Canada) automated liquid-handling instrument. RNA (1μg) was reverse transcribed into cDNA using the superscript VILO master mix kit (Invitrogen, Carlsbad, CA, USA). The cDNA was then used as template for Specific Target Amplification (STA) to enrich for targeted sequences and increase the sensitivity of the microfluidics platform. The 5 μl STA reaction contained 1.3 μl of cDNA/DNA, 1x TaqMan PreAmp master mix (Applied Biosystems, Foster City, CA, USA) and 0.2 μM of each of the primers (45 VDD host genes and 3 housekeeping genes run as singletons, 12 infectious agents run in duplicate; Table 2). The 14-cycle STA program followed manufacturer's instructions (Fluidigm Corporation, South San Francisco, CA, USA). Upon completion of the STA, excess primers were removed by treating with Exo-SAP-ITTM (Affymetrix, Santa Clara, CA, USA) according to manufacturer's instructions and then diluted 1/5 in DNA re-suspension buffer (Teknova, Hollister, CA, USA).

The 96.96 gene expression dynamic array (Fluidigm Corporation, CA, USA) contained TaqMan assays to both host genes and select infectious agent assays (Table 2) and generally followed Miller et al (2016). A 5-μL reaction mix [2x TaqMan Mastermix (Life Technologies), 20x GE Sample Loading Reagent, nuclease-free water and 2.7 μL of amplified cDNA] was added to each assay inlet of the array following manufacturer's recommendations. After loading the assays and samples into the chip by an IFC controller HX (Fluidigm), PCR was performed under the following cycling conditions: 50°C for 2 min, 95°C for 10 min, followed by 40 cycles of 95°C for 15 s and 60°C for 1 min. Gene expression data were preprocessed using GenEx (www.multid.se). Host biomarkers were normalized to the three reference genes, and relative gene expression was assessed using the 2^−ΔΔCt^ method (Livak and Schmittgen, 2001). A pooled sample was used as the relative control.

### Validation of proposed VDD panels

The discrimination capabilities of the resultant proposed VDD 45 biomarker panel were validated in independent fish from the jaundice syndrome, farm audit and wild salmon studies, and in samples from the IHNv challenge study that represented a mix of those previously used discovery analysis (13%) and new samples from waterborne experiments and additional tissues. For each dataset, the discriminatory capabilities of the full VDD biomarker panel was assessed using unsupervised PCA analysis and hierarchical clustering (heatmaps) based on Euclidean distance metric and complete linkage, with gene shaving (described above) applied to identify whether a reduced set of biomarkers carried similar discriminatory capabilities. To assess the contribution of individual biomarkers to discriminatory capabilities, two-sample *t*-tests between ‘viral diseased’ and either ‘healthy controls’ or ‘bacterial/parasitic diseased’ samples were used as implemented in the *t.test*-function in R's *stats* package (R version 3.3.1). Unequal variances were assumed and a Welch approximation to the degrees of freedom was used in the *t*-test. No multiple test correction was applied but a more stringent *P*-value threshold of 0.01 was used instead when assigning significance. Boxplots were generated to visually assess the degree and direction of differential expression between viral, bacterial and parasitic disease sample groups, with rectangles (boxes) representing the interquartile range (IQR) from the first quartile (the 25th percentile) to the third quartile (the 75th percentile) of the data. Whiskers extend from the box to the minimum and maximum value unless the distance from the minimum value to the first quartile is more than 1.5 times the IQR. In that case, the whisker extends out to the smallest value within 1.5 times the IQR from the first quartile and values outside the whisker are drawn as open circles. A similar rule is used for values larger than 1.5 times IQR from the third quartile (Krywinski and Altman, 2014). A final set of heatmaps based on all biomarkers applied across studies was generated to illustrate that VDD biomarkers were consistently up-regulated in fish in a viral disease state across studies. For each heatmap, samples were manually grouped into ‘healthy controls’, ‘viral diseased’ or ‘bacterial/parasitic diseased.’ For the farmed audit study, within-group samples were ordered by mean value over all biomarkers while for the IHNv validation data, non-control samples were ordered by day post-challenge. Genes were re-ordered among studies to highlight at the top the genes with the most consistent contribution.

Statistical analysis was performed with R version 3.2.1 (discovery) and 3.3.1 (validation) (R Core Team, 2016).

### Functional analysis of the VDD

Pathway Studio^TM^ (Elsevier, Amsterdam) was used to carry out functional analysis of the proposed VDD panel. VDD biomarker genes that could be annotated to mammalian genes were used in analyses to identify the most significant transcriptional regulator, over-represented biological and disease-related processes, and to develop a disease network, linking genes by their common regulators and closest neighbors.

## Results

### Microarray validation of discovery panels—candidate biomarker signatures

Of the 25 VRG SIQ features of Krasnov *et al.* (2011), 16 could be mapped to 71 unique GRASP16K features (using a gene name mapping approach based on a GRASP16 GPL annotation file). Fifteen of the remaining VRG features, not explored in our microarray analyses, were assessed via qRT-PCR.

Meta-analysis of signature CS0301u, a 532 feature panel derived from published microarray challenge studies for ISAv, IPNv, PMCv, PRv and IHNv, was conducted on in-house IHNv microarray studies across Atlantic, Sockeye and Chum salmon, yielding five signature panels (PBP016, PBP018, PBP19, PBP020 and PBP021) (Table S1). There are 54 features (some including gene paralogs) in the union of the five panels, including 15-feature panel PBP022 and 19-feature panel PBP024 which represent subsets of the Purcell *et al.* (2011) published signature, that were able to separate controls from exposed samples in MGL IHNv-challenged Sockeye and Atlantic salmon data. Visual inspection of boxplots for the 54 features in Atlantic, Sockeye, and Chum salmon, and Rainbow trout IHNv datasets, and selection of features that showed consistent behavior across species (maintained increase or decrease in expression after exposure), resulted in the selection of a subset of 38 features that define signature CPS301 (Table S2).

Signature CPS301 includes eight features and five genes from the VRG-signature in Krasnov *et al.* (2011): FRK (aka SRK2), IFIT5, RSAD2 (two features), CD9 (two features CD9 and two features Gig2-L), VIG10 (Table S1). In total, 6 of the 38 features in CPS301 were only found in the Rainbow trout and Chum data but they displayed strong differences in expression between controls and exposed samples in both species: Cox4nb, Zinc−binding protein A33, STAT1, unknown protein [*Siniperca chuatsi*] (CA038063), PRK12678 transcription termination factor Rho and RNF213.

All 38 features of CPS301 showed an increase of expression after exposure across species. Several of the features suggested transient increase of expression in Chum salmon, the species least susceptible to IHN, while increased expression was maintained in the other species for which there was data after pre-processing. IRF7 showed transient increase in expression in Atlantic salmon but a maintained increase in Chum salmon.

Two of the 38 features in CPS301 map to GIG2L (aka CD9), and in addition to being identified in Krasnov *et al.* (2011), they also show a clear change in expression in ISAv versus control samples in the LeBlanc *et al.* (2010) dataset, and in IHNv versus control samples in the Purcell *et al.* (2011) dataset (Table S1). Two additional features were found in the CPS301 signature and in the ISAv dataset: PLAUR and Slime mold cyclic AMP receptor, both being included in CPS301 as their expression suggested an involvement in a response to IHNv and ISAv-infection.

Additional features from Krasnov that could not be mapped to the GRASP arrays or that were often removed from testing due to data quality issues were added to the VDD validation panel, including DDX58 (two features; aka RIG1), BANF1, GVINP1, HERC4, IFI, IFIH1 (aka MDA5), IFI44 (two features), NFX1, RAD1, RTP3, SACS, VIG1, VIG4, XAF1 and ZFP9 (Table S2).

### VDD biomarker validation studies

The Fluidigm BioMark platform was used to validate 51 assays across 45 candidate VDD biomarker genes (including gene homologs). Note that not all assays showed high efficiency across all species; hence, for each species, we report only the assays with efficiencies between 0.85 and 1.1.

### IHNv challenges

VDD biomarkers were applied to 604 samples from IHNv challenge studies that included three salmon species (Atlantic, Sockeye and Chum), two types of challenges (ip injection and cohabitation) and multiple tissues (head kidney, gill, liver, spleen) sampled (Table 1A). Although at the outset of these experimental challenges fish were considered disease free, assays to 23 infectious agents applied simultaneous to the VDD biomarkers revealed a range of infectious agents detected across species, albeit most at low levels. The presence of these additional (bacterial and microparasite) infectious agents enabled the assessment of their impact on the resolution of fish with IHN, which was found to be minimal.

**Figure 2:**
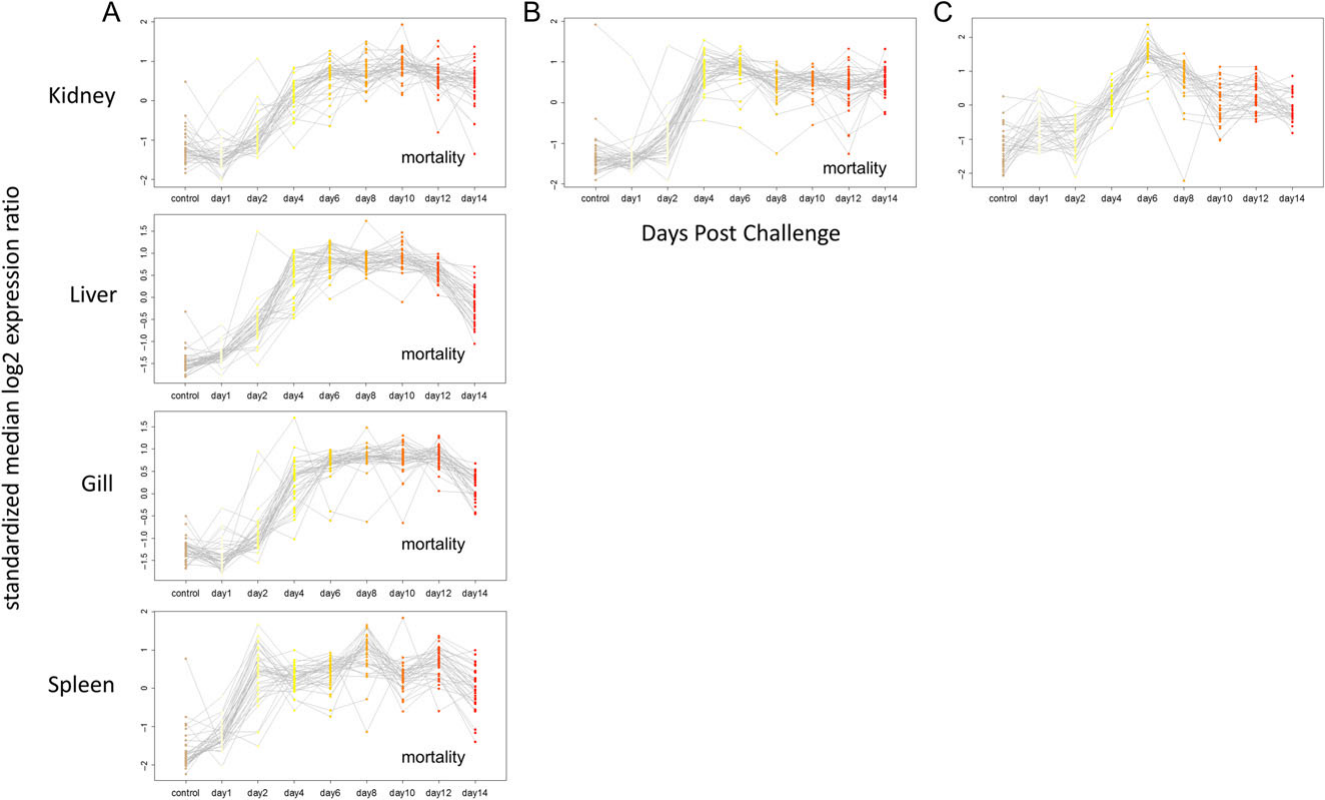
Time-course of expression of VDD genes post IHNv ip challenge, by tissue. (A) Sockeye, (B) Atlantic and (C) Chum salmon. Only samples from IHNv infected fish are included in the displayed post controls time course data. For Sockeye this included all 45 samples, while one Day 1 sample was excluded in the Atlantic salmon time course, and 11 samples from different time points were excluded in the Chum salmon time course.

**Figure 3:**
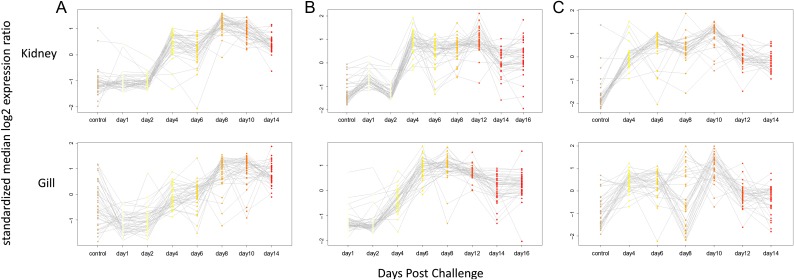
Expression of VDD genes on a time-course post IHNv waterborne challenge, by tissue. (**A**) Sockeye salmon (**B**) Atlantic salmon (**C**) Chum salmon. Post controls time course samples represent IHNv infected fish only (26 for Sockeye, 37 for Atlantic and 18 for Chum salmon). Only time points with data for at least two samples are displayed.

Sockeye salmon challenges included 275 samples assessed for VDD and infectious agents distributed across four tissues (head kidney, gill, liver and spleen) in the ip challenge and two tissues (head kidney and gill) in the waterborne challenge (Table 1A). Co-infections were extremely rare, affecting <7% of fish, none other than IHNv with loads exceeding 100 copies per μl. Microsporidian parasite *Paranucleospora theridion* was the only co-infecting agent affecting more than 5% of fish. Mortality reached 44% over the 30-day course of the ip challenge, starting on Day 9 and reaching 20% by Day 14, the last fish sample date. Transcriptional profiles of all IHNv positive fish against uninfected controls at Day 0 were averaged for each day post-infection. In every tissue across 38 of the 39 VDD biomarkers with good efficiencies in Sockeye salmon, a pattern of transcriptional up-regulation of ip challenged fish by Day 4 separated the IHN diseased fish with controls and early stage infections in all four tissues (kidney, liver, spleen, gill; Fig. 2A; Table 3). ZPF1 was the exception. Most biomarkers remained highly up-regulated through Day 12, many beginning to diminish by Day 14. A similar pattern was apparent in kidney and gill tissues from waterborne challenged fish that became infected with IHNv (Fig. 3A), although there was enhanced variability in gill tissue. While generally up-regulated, GNL3, VLIG, IFI35, CD68, CD9 (GIG2-1_MGB1 assay), PSMB8 and Trim21 showed enhanced variability over the time-course in some tissues (Table 3).

**Table 3:** Differential regulation of individual biomarkers within the VDD panel in response to IHNv challenges, by species, jaundice/anemia in Chinook salmon, and diseases on salmon farms. In IHNv and jaundice studies, ‘Up’ refers to up-regulation of biomarkers in IHN diseased versus control or early infection salmon and ‘Variable’ refers to biomarkers that do not show continuous up-regulation post-challenge. GS-VDD refers to biomarkers that were identified via gene shaving. Differential regulation in the farm audit studies in Atlantic and Chinook salmon was determined by expression box plots (top 11 presented in Figure 7). Biomarkers were ranked by overall discrimination capabilities with those classified as ‘Top’ performing consistently across all studies, ‘Good’ showing strong classification ability in most studies, ‘Limited V-B’ showing limitations in classifications between viral and bacterial diseases (not including bacterial kidney disease), and ‘Viral-Healthy’ only showing classification between viral-mediated diseased and healthy individuals

					IHNv challenge studies			Jaundice	Audit		Audit		Overall
Gene name	Assay name	Derived	Gene network	Gene ID	Sockeye	Atlantic	Chum	Chinook	Atlantic box	Chinook box	Atlantic	Chinook	
Ubiqitin-like protein-1, Peroxisomal membrane protein 2	UBL1_MGL_2	CPS301, Krasnov		PXMP2	Up	Up	Up Days 6–8	Up—GS-VDD_7	Excellent	Excellent*^bkd^	GS-VDD_9	GS-VDD_11	Top Overall and V-BKD^Chinook^
Interferon-induced protein with tetratricopeptide repeats 5	IFIT5_MGL_2	CPS301, Krasnov	Yes	IFIT5	Up	Up	Up	Up	Excellent	Excellent	GS-VDD_15		Top
Galectin-3-binding protein precursor	GAL3_MGL_2	CPS301		LGALS3BP	Up	Up	Up	Up	Excellent	Excellent*^bkd^		GS-VDD_11	Top
Zinc finger NFX1-type	NFX_MGL_2	Krasnov		NFX1type	Up	Up	Variable	Up	Excellent	Excellent			Top
VHSV-inducible protein-4	VHSVIP4_MGL_3	Krasnov		VIG4	Up	Up	Up Days 6–8	Up	Excellent	Excellent	GS-VDD_9	GS-VDD_9	Top
ATP-dependent RNA helicase	DEXH_MGL_3	Krasnov-not 16K	Yes	DDX58		Up			Excellent	Excellent*^loma^			Top
Interferon-induced GTP-binding protein Mx	MX	in house	Yes	MX1	Up	Up	Up	Up—GS-VDD_7	Excellent	Excellent	GS-VDD_9	GS-VDD_11	Top
Radical S-adenosyl methionine domain-containing protein 2	RSAD_MGB2	CPS301, Krasnov	Yes	RSAD2	Up	Up	Up	Up	Excellent	Excellent	GS-VDD_9	GS-VDD_9	Top
Mitochondrial ribosomal protein (VAR1)	CA054694_MGL_1	CPS301		VAR1	Up	Up		Up	Good	Excellent	GS-VDD_9		Top
IFN-induced protein 44-1	IFI44A_MGL_2	Krasnov-not 16K		IFI44	Up	Up		Up	Good	Excellent*^bkd^	GS-VDD_15		Top and BKD^Chinook^
Probable E3 ubiquitin-protein ligase HERC6	HERC6_1	CPS301	Yes	HERC6	Up	Up	Up	Up—GS-VDD_7	Good	Excellent^*loma^	GS-VDD_15		Top
52 kDa Ro protein-2—52Ro	52RO_MGL_3	Krasnov	Yes	TRIM21	Variable		Up	Up	NA	Excellent		GS-VDD_9	Top Pacific
IFN-induced protein	IFI_MGB2	Krasnov			Up	early, Variable	Up Days 4–8	Up	NA	Excellent		GS-VDD_11	Top Pacific
CD9 molecule	GIG2-1_MGB3	CPS301, Krasnov	Yes	CD9		Up			Excellent	NA	GS-VDD_9		Top Atlantic
Retinoic acid-inducible gene-1	RAD_MGL_2	Krasnov		RAD1		Up			Excellent	NA			Top Atlantic
Sacsin	SAC_MGB2	Krasnov		SACS		Up			Excellent	NA	GS-VDD_9		Top Atlantic
XIAP-associated factor-1	XAF1_MGL_1	Krasnov-not 16K	Yes	XAF1		Up			Excellent	NA			Top Atlantic
Receptor-transporting protein 3	RTP3_MGL_1	Krasnov		RTP3		Up			Excellent	Good	GS-VDD_9		Good
Signal transducer and activator of transcription 1-alpha/beta	STAT1	CPS301	Yes	STAT1	Up	Down early	Up Days 4–8	Up	Good	Poor		GS-VDD_9	Good
Tyrosine-protein kinase FRK	SRK2_MGB3	CPS301, Krasnov		FRK	Up	Up			Good	Good		GS-VDD_11	Good
*Oncorhynchus mykiss* VHSV-induced protein-10	VHSV-P10_MGL_2	CPS301		VIG10	Up	Up	Up Days 4–8	Up—GS-VDD_7	Good	Poor		GS-VDD_11	Good
ATP-dependent RNA helicase	DEXH_MGL_1	Krasnov-not 16K	Yes	DDX58	Up		Up	Up	NA	NA			Good
RNA helicase—RIG-I	RIG1_MGLSYBR_1	Krasnov	Yes	DDX58	Up	Up	Up	Up—GS-VDD_7	Excellent	Good^*loma^			Good
Very large inducible GTPase 1-1	VLIG1-1_MGL_2	Krasnov		GVINP1	Variable	Up early	Variable	Up	Poor	Good^*loma^			Good
Similar to KIAA1593	KIAA_MGL_1	Krasnov-not 16K		HERC4	Up		Up Days 1–14	Up	NA	NA			Good
MHC class I antigen [*Salmo salar*]	UBA_MGL_CA050178_1	CPS301		MHC1		Up			NA	NA			Good
MHC class I antigen [*Salmo salar*]	UBA_MGL_CA050178_4	CPS301		MHC1	Up				NA	NA			Good
Ring finger protein 213	E3RNF213_2	CPS301		RNF213	Up	Up	Up Days 4–10		Poor	Excellent^*loma^			Good
Ring finger protein 213	RNF213_MGL_5	CPS301	Yes	RNF213	Up	Up	Up	Up	Good	Poor			Good
Sacsin	SAC_MGB1	Krasnov		SACS	Up			Up	Excellent	NA			Good
Ubl carboxyl-terminal hydrolase 18	USP18_MGL_2	CPS301	Yes	USP18	Up	Up	Up Days 4–8	Up—GS-VDD_7	Excellent	Good			Good
VHSV-inducible protein-1	VHSVIP1_MGL_3	Krasnov-not 16K		VIG1	Down early		Up Days 4–8	Up—GS-VDD_7	NA	NA			Good
XIAP-associated factor 1	XAF1_MGL_2	Krasnov-not 16K	Yes	XAF1				Up	NA	NA			Good
Barrier to autointegration factor	BAF_MGL_4	Krasnov		BANF1		Up			Good	NA	GS-VDD_9		Good Atlantic
CD9 molecule	GIG2-1_MGB2	CPS301, Krasnov	Yes	CD9			Up	Up	NA	NA			Good Pacific
IFN-induced protein 44—IFI44	IFI44C_MGL_3	Krasnov		IFI44	Up		Up	Up	Not Rickettsiosis	Excellent*^bkd^	GS-VDD_15		Good
Urokinase plasminogen activator surface receptor precursor	PLAUR_MGL_3	CPS301	Yes	PLAUR	Up	Up	Up	Up	No Discrimination	Excellent	GS-VDD_15	GS-VDD_9	Limited V-B_Atlantic_
PLAC8-like protein 1	PLAC8L1_MGL_1	CPS301		PLAC8L1	Up	Up	Up	Up	Good	Poor		GS-VDD_9	Limited V-B_Chinook_
unknown protein [*Siniperca chuatsi*]	CA038063_MGL_1	CPS301				Up		Up (not gill)	No Discrimination	BKD not Loma			Limited V-B but Viral-BKD^Chinook^
CD68 molecule	CD68_MGL_3	CPS301	Yes	CD68	Variable	Up	Up	Up	Only Mouth Rot	Poor			Limited V-B
CD9 molecule	CD9_MGL_2	CPS301, Krasnov	Yes	CD9	Up	Up	Up	Up	Not Rickettsiosis	Good*^loma^		GS-VDD_9	Limited V-B
Slime mold cyclic AMP receptor	DICTY.CAR_MGL_2	CPS301		DICTY	Up	Up	Up Days 1–14	Up	Only Vibriosis	Poor		GS-VDD_11	Limited V-B
Interferon-induced 35 kDa protein homolog	IFI35_MGL_2	CPS301		IFI35	Variable	Up		Up	Only Rickettsiosis	Poor		GS-VDD_9	Limited V-B
Interferon induced with helicase C domain 1—IC	IFI-1C_MGL_2	Krasnov	Yes	IFIH1	Up	Up	Variable	Up	Poor	Poor			Limited V-B
Interferon regulatory factor 7	IRF7_MGL_2	CPS301	Yes	IRF7	Up	Up	Up	Up	Not Winter Ulcer	Good^*loma^	GS-VDD_15		Limited V-B
Guanine nucleotide-binding protein-like 3	GNL3_MGL_1	CPS301		GNL3	Variable	Up early, Variable	Variable	Up	No Discrimination	No Discrimination		GS-VDD_9	Limited V-B
Proteasome subunit beta type-9 precursor	PSMB9A_MGL_2	CPS301	Yes	PSMB9	Up	Down early	Up	Up	No Discrimination	Good			Limited V-B
similar to interferon-inducible protein Gig2	GIG2-1_MGB1	CPS301, Krasnov	Yes	CD9	Variable								Poor
Fish virus induced TRIM-1	TRIM1_MGB1	Krasnov-not 16K		TRIM1	Up	Variable	Variable	Up	Poor	Poor			Poor
Proteasome subunit beta type-8 precursor	PSMB8_1	CPS301	Yes	PSMB8	Variable		not sig	Up (not gill)	Poor	Excellent*^bkd^			Poor but V-BKD^Chinook^
PR domain zinc finger protein 9	ZFP9_MGL_2	Krasnov		ZFP9	None	Down	None	None	NA	NA			Poor

**Figure 4:**
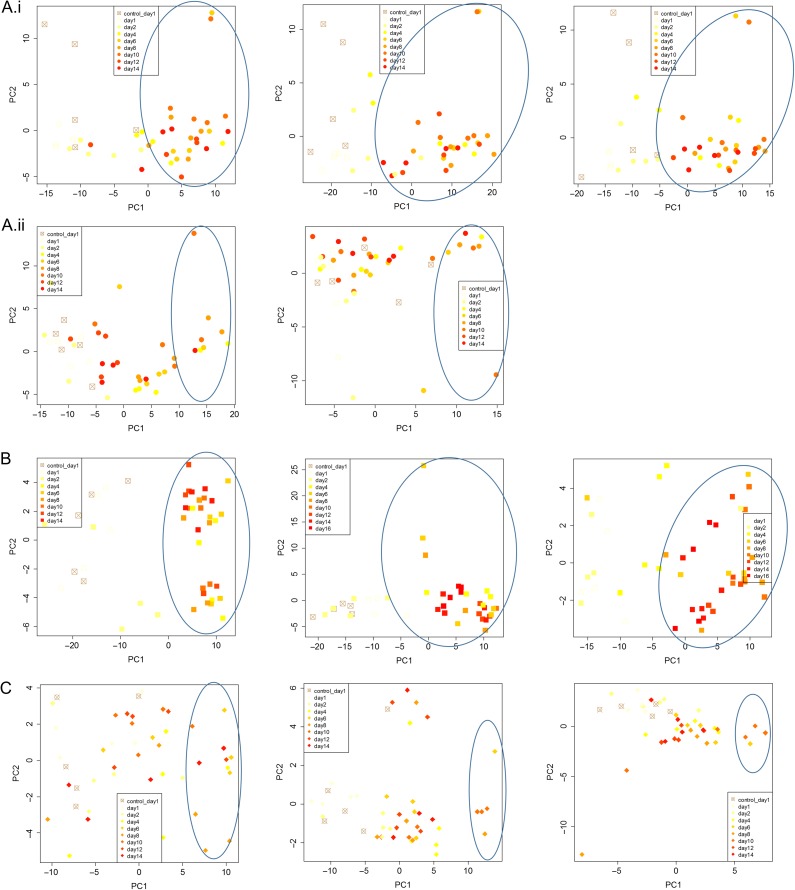
PCA classification of salmon post IHNv challenge, by species, challenge-type and tissues, as visualized by principle component analysis. (**A**) Sockeye salmon: (i) ip-challenge by tissue (head kidney, liver, gill, respectively) and (ii) waterborne-challenge by tissue (head kidney, gill, respectively). (**B**) Atlantic salmon by challenge-type and tissue (ip: kidney, waterborne: head kidney, gill, respectively). (**C**) Chum salmon by challenge-type and tissue (ip: head kidney, waterborne: head kidney, gill, respectively).

For the Sockeye salmon ip challenge, discrimination of fish with IHN was high from Day 4 onward (Fig. 4A(i)). IHNv abundance quickly elevated in the spleen, which also showed earlier development of the VDD, a few fish even on Days 1 and 2 post ip injection, however, the opposite pattern was observed later on. From Days 6 to 12, all but one fish was in a VDD state across tissues, the exception being a fish with very low IHNv detection on Day 12. IHNv loads and VDD strength diminished on Day 14, with head kidney the most impacted. In the waterborne challenge, only a small number of fish became infected in the head kidney, and stronger loads were generally detected in the gill. Only fish with detectable IHNv across gill and head kidney were in a VDD state across tissues; five fish with very low load detections across tissues did not classify as VDD, and one fish with moderate loads in gill and low loads in head kidney classified as VDD only in gill tissue (Fig. 4A(ii)). There were no measurable impacts of co-infective agents on discrimination of IHN fish in either challenge.

Atlantic salmon challenges included 138 samples assessed using the VDD panel and infectious agents across two tissues (head kidney and gill) (Table 1A). Co-infecting agents affecting >5% of fish included *P. theridion* (25%) and *Flavobacterium psychrophilum* (6%). Mortality reached 100% over the 30-day course of the ip challenge, starting on Day 9 and reaching 75% by Day 14, the last fish sample date. Transcriptional up-regulation was synchronous among 40 of the 41 VDD biomarkers with good efficiencies in Atlantic salmon from Days 4 to 14, in both head kidney and gill tissues (Fig. 2B). VLIG1, GNL3 and IF1 were up-regulated earlier, starting on Day 2 (Table 3). ZPF9 was the only VDD biomarker that was down-regulated, also starting Day 4. TRIM1, although up-regulated, showed high variability post-challenge. These gene transcriptional patterns were highly consistent in the gills of waterborne challenged fish that became positive for IHNv, although most biomarkers peaked in transcription on Days 8–10 and then showed slight down-regulation on Days 14–16 (Fig. 3B), only a couple of which (PSMB9 and TRIM1) dropped to pre-exposure levels. In head kidney, GNL3 and IF1 showed higher variability, and three genes, PLAUR, PSMB9 and STAT1, were down-regulated on Days 14 and/or 16 (Table 3). ZFP9 was not consistently affected. Discrimination of fish with IHN was high from Day 4 onward (Fig. 4B).

Chum salmon challenges were conducted over 133 samples, including head kidney from an ip challenge and head kidney and gill from a waterborne challenge (Table 1A). In addition to the co-infective agents in Sockeye and Atlantic salmon, 63% of fish contained myxozoan parasite *Parvicapsula pseudobranchicola*, some at well over >100 copies per μl. Mortality of Chum salmon was <10% for each challenge study, and IHNv was detected in only 72% of ip-challenged fish and 38% of waterborne-challenged fish (across tissues), although only 18% in head kidney. Although IHNv copy number reached 10^5^ in a few ip-challenged fish, individual levels varied dramatically from 10^1^ to 10^4^ on any given sample day, but were most consistent, with an average of 10^4^, on Days 6–8. Transcriptional up-regulation in response to IHNv ip challenge of IHNv positive fish followed patterns of peak IHNv loads, generally occurring between Days 4–8 and involving fewer biomarkers than in Sockeye and Atlantic salmon (Fig. 2C). Of the 34 biomarkers assessed with good efficiencies in Chum salmon, 28 were consistently up-regulated in head kidney samples from the ip challenge, eight with limited duration (Table 3). HERC4 and DICTY were up-regulated earlier than other biomarkers, from Day 1 onward.

In the Chum salmon ip challenge, PCA analysis showed VDD clustering mostly contained to fish with IHNv loads >10^3^ in head kidney, although a few fish on Days 2–4 with lower IHNv loads, and one with no IHNv detection in head kidney, were also within this cluster (Fig. 4C). In the waterborne challenge, IHNv was weakly detected in one fish on Day 1 in gill tissue, but was not detected until Day 6 in the head kidney, and even then, only in a single fish. Copy numbers of IHNv never reached more than 10^2^ in gill, but reached 10^4^ in the head kidney of one fish. IHNv was detected in head kidney in 40% of fish sampled on Days 6–10 and then dissipated. Transcriptional up-regulation of VDD biomarkers and VDD classification in gill and head kidney was restricted to fish with IHNv detections >10^3^ in head kidney tissue (Fig. 4C); detections in gill alone did not elicit a substantive response. Similar genes as observed in the ip challenge were up-regulated.

### Jaundice syndrome

Jaundice syndrome (aka jaundice/anemia) is a disease causing low level mortality in farmed Pacific salmon with a suspected viral etiology, but that has not undergone extensive study. We explored the performance of the VDD biomarkers on fish undergoing natural farm outbreak of jaundice syndrome to further assess the likely viral etiology of the disease, and to quantitatively assess 45 salmon infectious agents for association with jaundice/anemia. Five tissues (liver and anterior kidney—the tissues showing most necrotic damage, heart, gill and spleen) were each assessed across 36 Chinook salmon, including diseased and healthy controls (Table 1B). Being a natural outbreak, there was a high rate of co-infection in these fish, with nine infectious agents detected, six at high loads in some individuals [up to 10^5^ per μl—PRv (90% prevalence), *Candidatus Branchiomonas cysticola* (36%), *P. theridion* (64%), *Loma salmonae* (37%); 10^4^ per μl—*P. pseudobranchicola* (20%), *Renibacterium salmoninarum* (32%)]. PRv was present at high load in virtually all jaundice fish, with control fish negative or containing only low loads. PRv was the only infectious agent statistically correlated, by presence and load, with the disease (*R*^2^: 0.76–0.84 among tissues).

There were 40 VDD biomarkers that amplified at high efficiency in Chinook salmon; all but ZFP9 and unknown CA068063 were up-regulated in jaundice fish relative to controls, regardless of co-infecting agents or tissue. PSMB9, IFI44 (assay IFI44A_MGL_2) and VSVP10 showed more variability in control fish (Table 3).

**Figure 5:**
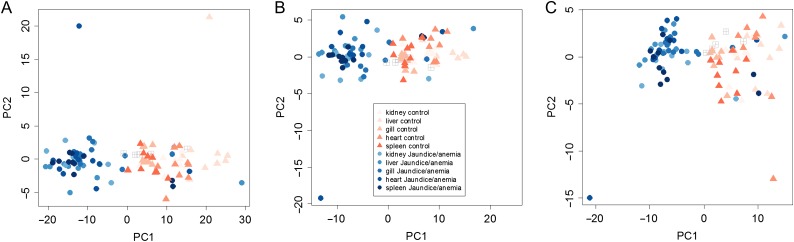
PCA classification of five tissues from farmed Chinook salmon undergoing an outbreak of jaundice/anemia. Analysis based on (**A**) a 40 biomarker VDD panel, (**B**) a 17 biomarker VDD panel identified through gene-shaving and (**C**) a 7 biomarker VDD panel derived from gene shaving. In each plot, samples with viral jaundice are shown in blue and healthy controls in peach, with shades and shapes within each depicting different tissues, as illustrated in the panel legend under (B). The single viral jaundice sample not properly classifying showed weak lesions and low viral loads, and is suspected to represent a fish in recovery.

There was near perfect separation of jaundice from healthy fish based on the 40 VDD markers across liver, anterior kidney, heart and gill tissues (Fig. 5). The single outlier across all tissues was a fish with anemia (not jaundice) that had weak histopathological lesions consistent with jaundice syndrome but did not contain high loads of PRv; this fish clustered with the ‘healthy’ controls. A second sample with jaundice did not classify correctly in spleen tissue (Fig. 5B). Gene shaving applied to kidney and liver reducing the VDD panel down to 22 biomarkers, and then to seven, produced equivalent separation of groups (Fig. 5). The top seven features identified through gene shaving were PXMP2, HERC6, MX1, USP18, VIG1, DDX58 (RIG1) and VIG10.

### Farm audit samples

VDD biomarkers were tested on combined tissues of 240 moribund/recently dead farmed Atlantic salmon and 68 farmed Chinook salmon collected through a regulatory farm audit program (Table 1C). Because our previous two validations showed that the VDD panel had discriminatory capabilities across tissues, we reasoned that this panel may still work effectively in combined tissue samples. Histopathology and clinical data had been applied previously to diagnose known, well characterized diseases, and qRT-PCR data performed across 49 infectious agents were available to identify known pathogens and validate these diagnoses. The application of the VDD panel to the audit samples offered perhaps the most complex co-infection scenario imaginable as dying fish are likely the most vulnerable to opportunistic pathogens. Most samples contained mixed infections with 2–10 agents identified per individual. Only two viruses were commonly observed across samples, PRv (69%) and erythrocytic necrosis virus (ENv) (21%), a DNA virus that causes erythrocytic inclusion body syndrome (EIBS). Unfortunately we did not have the blood smears to diagnose EIBS, so this disease was left off of our differentials.

**Figure 6:**
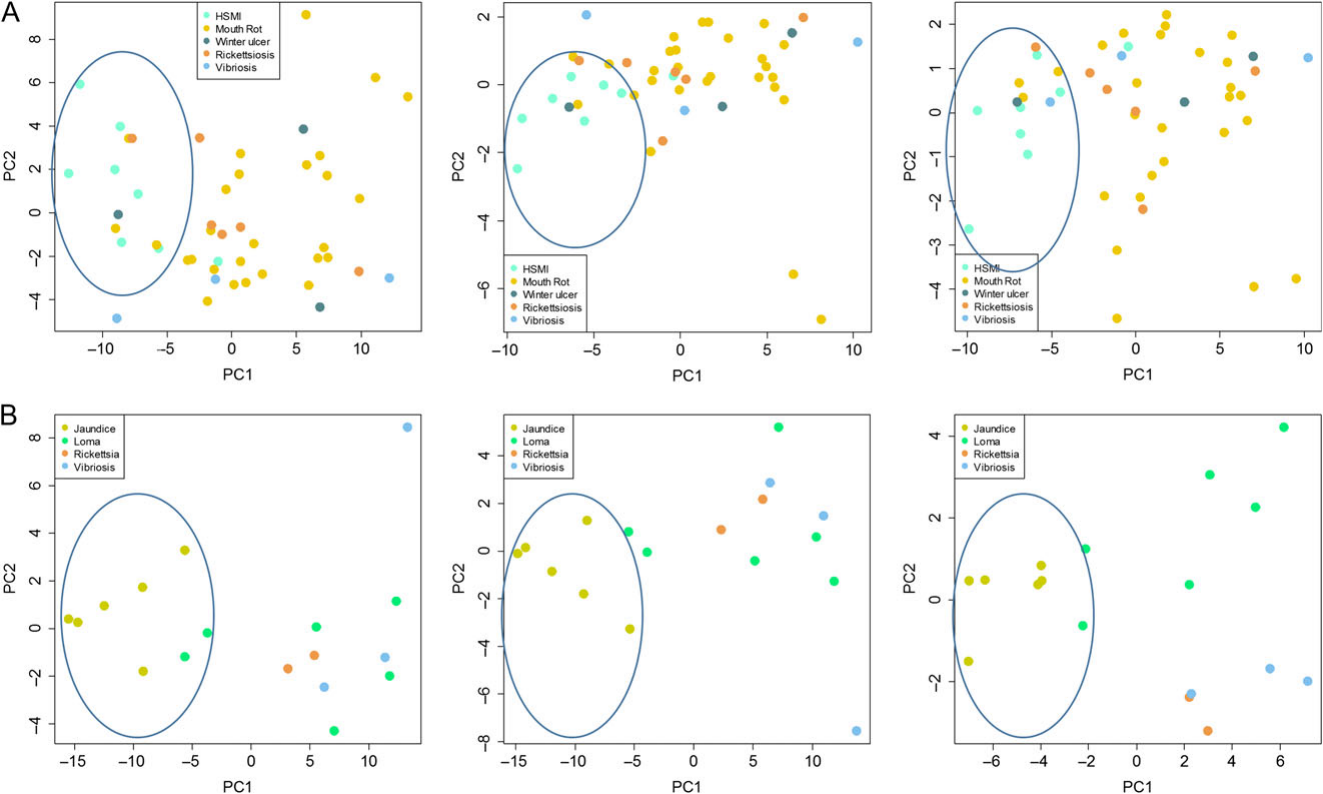
Principle component analysis depicting the differentiation of (**A**) Atlantic salmon farm audit fish (based on mixed tissue cDNA) diagnosed with viral (HSMI) versus bacterial diseases (mouth rot, winter ulcer, rickettsiosis, and vibriosis) based on the full 40 biomarker VDD panel (left), 15 biomarker VDD panel (mid) and 9 biomarker VDD panel (right) derived from gene shaving. (**B**) Chinook salmon farm audit fish diagnosed with viral (jaundice/anemia) versus bacterial (rickettsiosis and vibriosis) and parasitic (*Loma*) diseases based on the full 36 biomarker VDD panel (left), 25 biomarker VDD panel (mid), and 9 biomarker VDD panel (right) derived from gene shaving.

**Figure 7:**
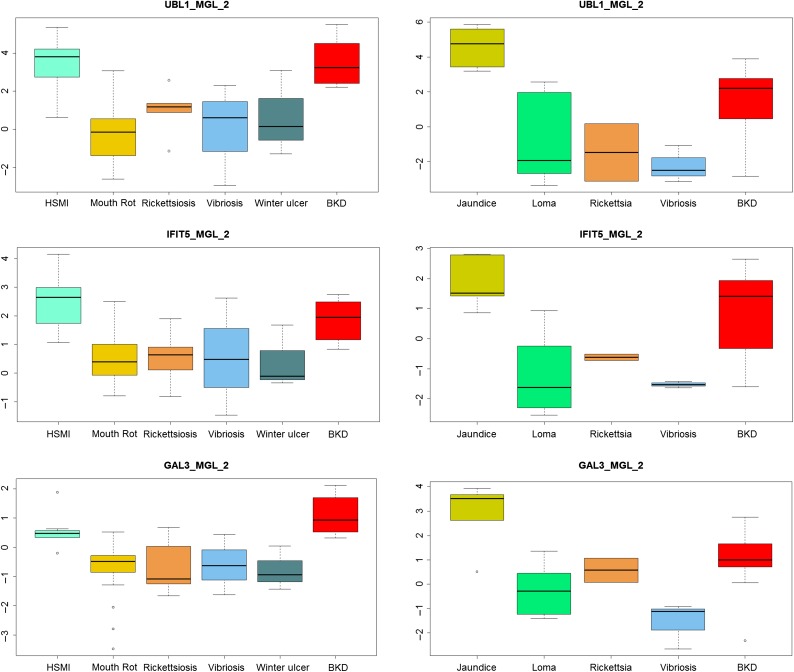

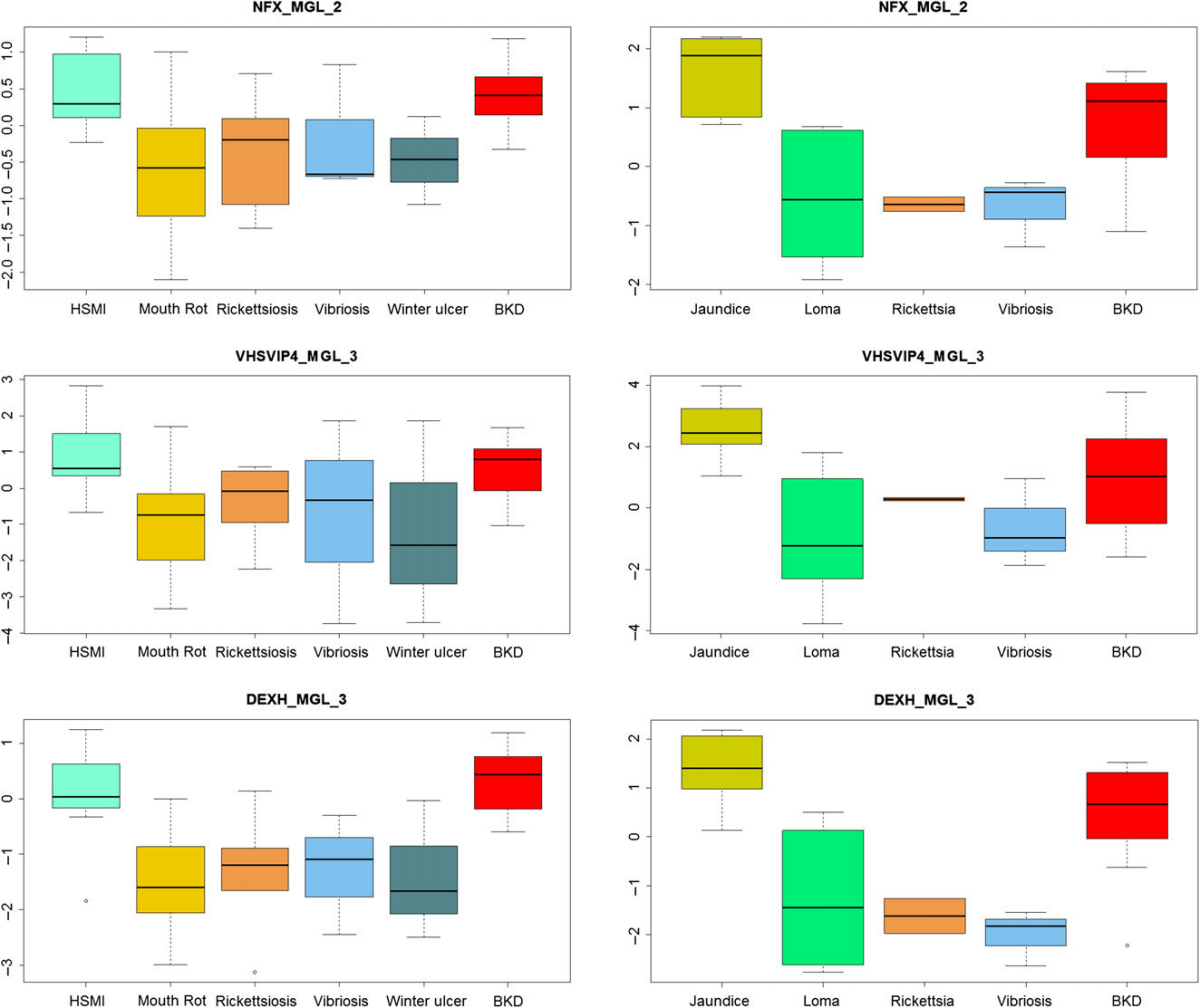

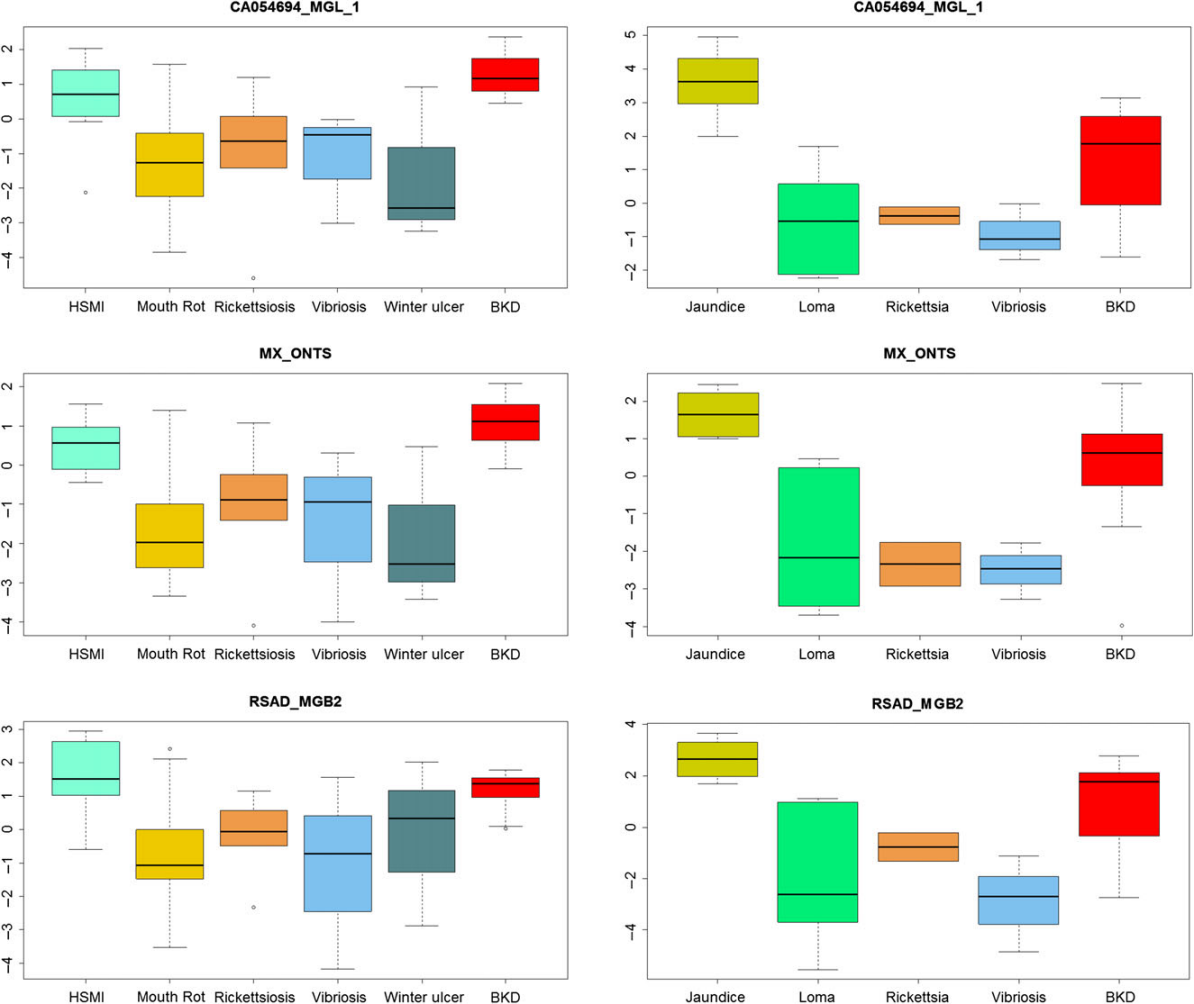

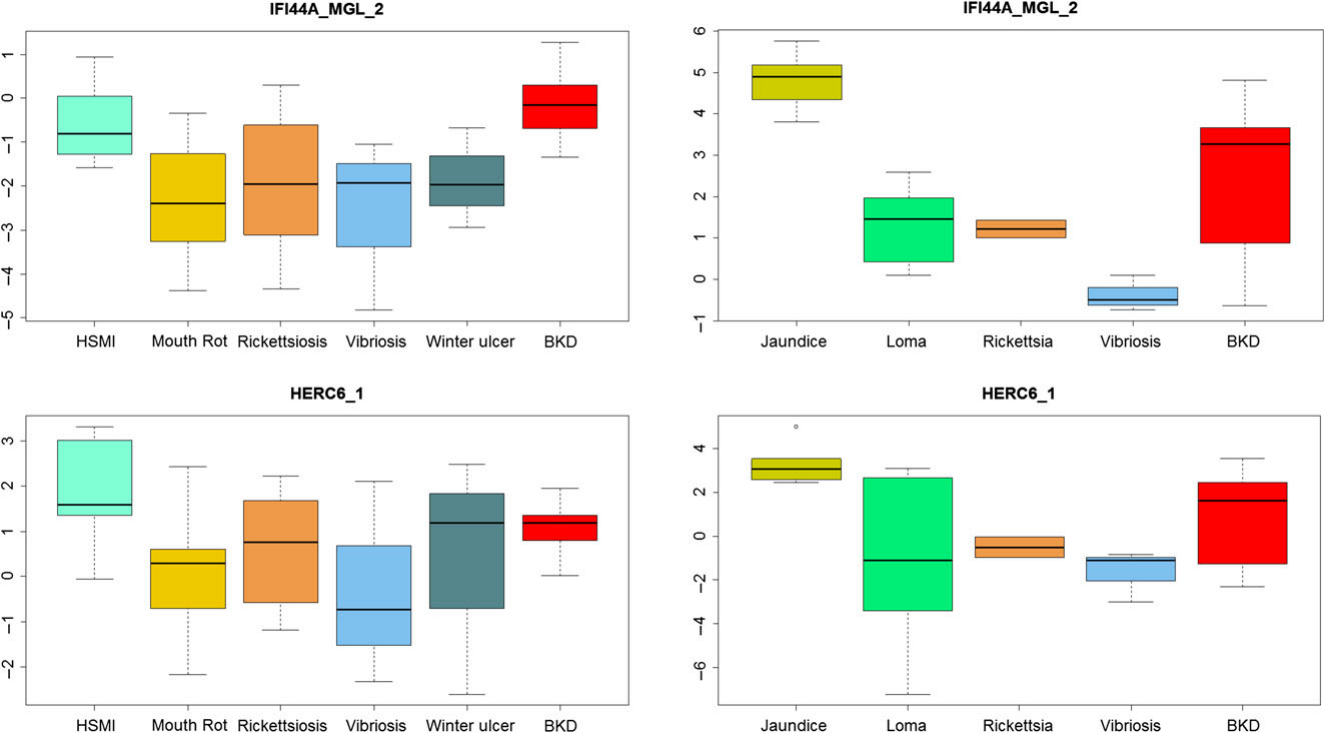
Gene expression box plots of top 11 biomarkers in the VDD panel for Atlantic (left) and Chinook (right) salmon from the farm audit study, contrasting median expression levels between viral (HSMI or jaundice) and bacterial (rickettsiosis, vibriosis, mouth rot, winter ulcer, and bacterial kidney disease [BKD]) or parasitic (*Loma*) diseases.

Overall, 30% of Atlantic salmon and 50% of Chinook salmon were diagnosed to a specific disease as the cause of death, confirmed by molecular detection of the etiological agents associated with pathologically identified diseases. VDD panel validations to differentiate viral from bacterial and parasitic diseases were based only on samples with specific diagnoses. Commonly diagnosed infectious diseases in farmed Atlantic and Chinook salmon included those caused by bacterial agents [rickettsiosis, bacterial kidney disease (BKD), mouth rot, vibriosis and winter ulcer], parasitic agents (*Loma*) and viral agents [heart and skeletal muscle inflammation (HSMI), jaundice/anemia]. We applied unsupervised PCA to the VDD panel datasets containing samples with HSMI (Atlantic) or jaundice/anemia (Chinook) and each bacterial or parasitic disease, removing fish diagnosed with bacterial or parasitic diseases that also carried PRv loads >100 copies per μl, the agent associated with both viral diseases. This cut-off was determined empirically as the approximate lower load limit of PRv associated with either viral disease. In each case, PC1, which explained 63% of the variation in Atlantic salmon and 78% in Chinook salmon, differentiated fish with viral versus bacterial/parasite infections (Fig. 6). In Atlantic salmon, there was a single HSMI outlier that was not tightly contained within the PC1-negative ‘viral’ cluster (roughly defined by PC1 loading <−5), but was still negatively loaded (−1); this fish had only weak heart lesions and carried moderate loads of PRv (10^2^ copies; possibly a recovery fish) (Fig. 6). There were also occasional fish diagnosed with bacterial diseases that clustered as ‘VDD.’ In Atlantic salmon, this included 3 of 24 fish with mouth rot, one of three fish with winter ulcer, one of five fish with rickettsiosis, and one of three fish with vibriosis. In Chinook salmon, two of the six fish with *Loma* clustered at the margins of the jaundice VDD samples. As in each case, these fish were outliers to the other samples under the same diagnostic category, we suspected that they may carry a co-infection with a virus that was not on our panel or an uncharacterized strain of PRv that our assay did not detect; high throughput sequencing is being pursued on these samples, with novel viruses already identified (K. Miller unpublished data). The only viral-bacterial contrasts that did not consistently yield strong differentiation with viral disease samples were those involving BKD (Fig. 7), a bacterial disease caused by the intracellular bacterium *R. salmoninarum*.

Gene shaving was applied to the above PCAs for Atlantic and Chinook salmon to determine whether a smaller VDD panel would yield similar separation between viral and bacterial diseases, which would increase the practicality of the VDD biomarker approach for routine diagnostic applications. We were able to obtain similar patterns of separation with a VDD panel comprised with as few as nine biomarkers (Fig. 6 and Table 3).

There was more variance in the contribution of individual biomarkers to classify viral versus bacterial/parasitic diseases in the audit samples than observed to distinguish viral disease from healthy individuals in other studies (Fig. 7). However, over half of the VDD biomarker assays showed strong separation between viral disease and all bacterial diseases tested, except BKD, and most of these also differentiated viral disease from disease associated with the microsporidian parasite *Loma* (Table 3). In Atlantic salmon, 17 of the 40 VDD biomarkers showed strong differential regulation between HSMI and the four bacterial diseases, and another eight were moderately up-regulated in viral versus bacterial diseases. In Chinook salmon, 14 of the 36 VDD biomarkers were powerfully up-regulated under viral jaundice compared to the three bacterial diseases, nine moderately so; six of these markers did not show the same level of discrimination between viral jaundice and Loma infection. Unlike for Atlantic salmon, for which none of the biomarkers showed particular discrimination between viral disease and BKD, in Chinook salmon, five biomarkers—UBL1 (PSMP2), LGAL3BP (GAL3), IFI44 (both A and C paralogs) and PSMB8—were discriminatory. Over both species, the strongest biomarkers for discrimination between viral and bacterial/parasitic disease were UBL1 (PXMP2), IFIT5, GAL3, NFX, VHSVIP4 (VIG4), DEXH (DDX58), unknown CA054694 MX1 RSAD, IFI44A, and HERC6 (Fig. 7 and Table 3).

### Wild migrating salmon

We applied the VDD panel assays on cDNA from gill biopsy samples (comprised of the tips of 1–2 gill filaments) sampled across 213 wild-migrating Sockeye salmon. Given the size of the samples (some not much larger than a pin head), we recognized the possibility that there may be false negative detections of infectious agents. Ten infectious agents were detected by Ct <27 (the average limit of detection on the BioMark platform; Miller *et al.* (2016)) in the gill tissue samples, including three bacteria—*C. B. cysticola*, *F*. *psychrophilum* and rickettsia-like organism; four parasites—*Ceratomyxa shasta*, *Ichthyophthirius multifiliis*, *L. salmonae *and *Myxobolus arcticus*; and three viruses—IHNv, PRv and Pacific salmon parvovirus (PSPv). *C. B. cysticola* was the only highly prevalent agent, detected in 86% of samples, with *F. psychrophilum* detected in 17% of fish. All other agents were detected in <8% of fish. Of the viruses, only IHNv was observed at appreciable copy number (>100 copies per μl).

**Figure 8:**
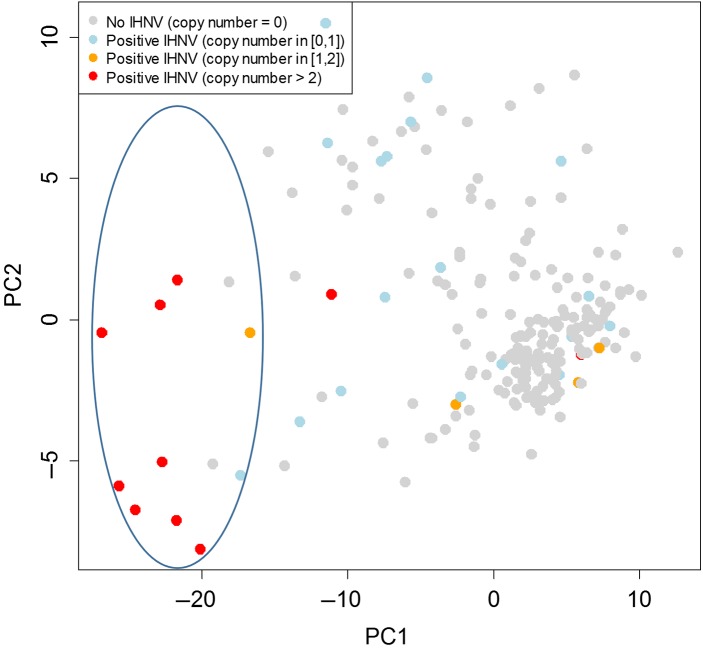
Principle component analysis of 39-biomarker VDD panel applied to non-destructive gill tissue from 213 wild migrating Sockeye salmon smolts. Coloring depicts VDD separation of most fish carrying high IHNv loads (log copy number >2).

PC1 of the 39 biomarker VDD panel strongly segregated fish with high IHNv loads, and explained 53% of the overall transcriptional variation in the data (Fig. 8). Overall, 9 of 10 fish with high IHNv loads clustered on the negative end of PC1. Within the distal ‘high load IHNv cluster’, there were also a small number of samples with moderate (1) and low (4) IHNv loads, as well as IHNv negative (7), but this represented a minority (<10%) of the samples overall. Whether these samples may carry an uncharacterized RNA virus is not known.

### Functional analysis of the VDD

**Figure 9:**
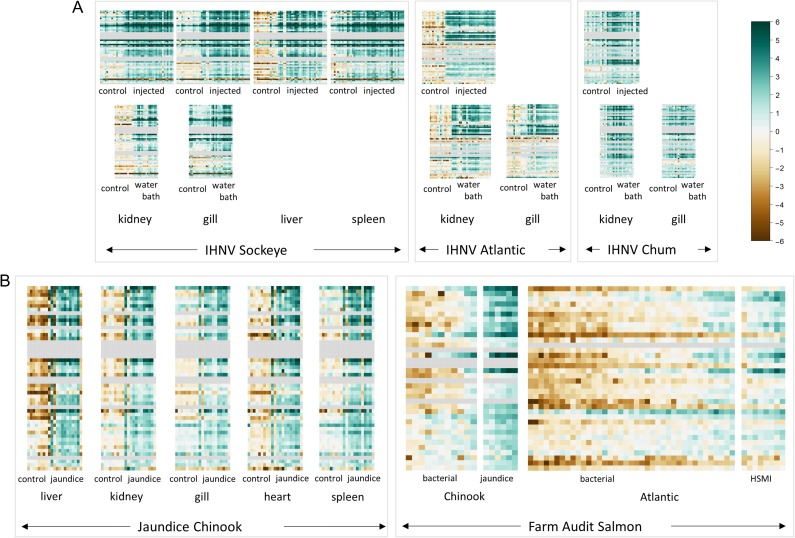
Heatmaps for the IHNv and farm audit validation datasets showing the up-regulation of VDD biomarkers in fish tissues under viral challenge (**A**) and in natural disease outbreaks (**B**). Heatmap depicting relative gene express (2^−ΔΔCt^ method) is scaled from brown (down-regulated) to teal (up-regulated) with darker colors indicating higher expression differentials as indicated by the color key on the top right. Grey rows indicate that no working assay was available for the corresponding genes. The Sockeye, Atlantic and Chum IHNv datasets depicted in (A) include heatmaps for multiple tissues (head kidney, gill, liver and spleen) from fish that were injected with IHNv (top), exposed to IHNv in waterbath (bottom), and controls that were not injected or exposed (both). The Jaundice Chinook dataset (B, left) includes heapmaps for head kidney, gill, liver, heart and spleen samples and Farm Audit Salmon datasets (B, right) show heatmaps for mixed-tissue samples.

**Figure 10:**
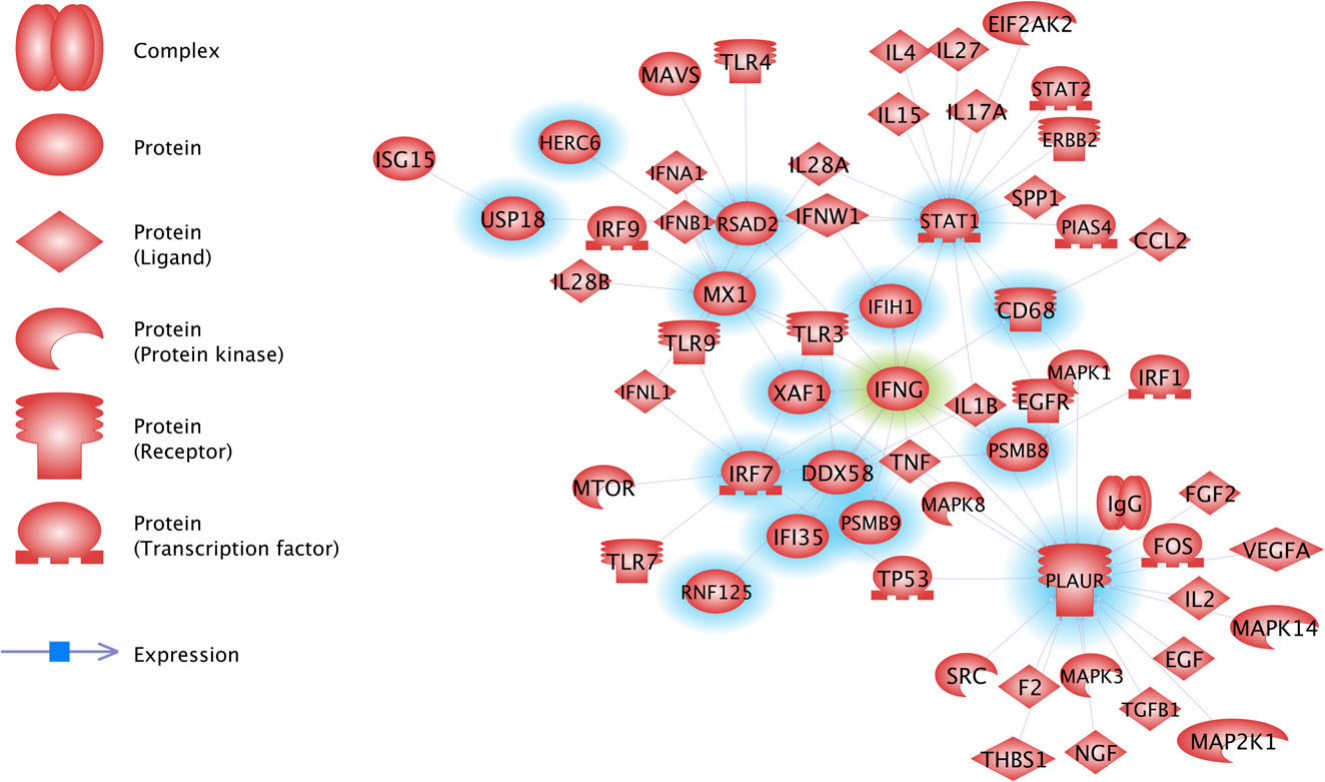
Gene network constructed based a 27-gene VDD panel identified to mammalian genes based on their gene symbols, showing key regulators (IFNG highlighted in green) and nearest neighbors (no highlight) overlaid onto the cell to show localization of protein activity. Analysis performed in Pathway Studio (Elsevier, Amsterdam). Fourteen VDD genes mapping to mammalian genes (IFH1, HERC6, RSAD2, DDX58, CD9, MX1, IFIT5, STAT1, XAF1, MX1, RNF213, TRIM21, USP18) are found within this gene network (highlighted in blue).

While the VDD biomarkers varied somewhat in the strength and consistency of their differential regulation associated with various viral diseases, where significant, they showed a consistent pattern of up-regulation in fish in a viral disease state (Fig. 9). Overall, 42 of the 45 unique VDD biomarkers could be identified to mammalian genes based on their gene symbols. IFNG was identified as the most significant regulator of the VDD panel (*P* = 5.45E-61; Fig. 10). Within the VDD panel, STAT1, IRF7 and DDX58 were also significant transcriptional regulators. Top significant (*P* < 0.001) diseases associate with the gene panel included ‘virus diseases’, ‘viremia’, ‘infection’, ‘HIV-1 infection’ and ‘inflammation’, and significant disease collections, as depicted by Pathway Studio^TM^, included ‘Dendritic Cell Activation in Systemic Lupus Erythematosus’ and ‘MAVS in Antiviral Innate Immune Response in Myocarditis’. Top cell processes included ‘response to viruses’, ‘viral reproduction’, ‘virus expression’, ‘protein ubiquination’, ‘innate immune response’, ‘response to dsRNA’ and ‘adaptive immune response’. The top immunological pathway was ‘Antiviral Signaling through Pattern Recognition Receptors’.

## Discussion

Meta-analysis of multi-cohort data identified gene-mapped microarray features that were consistently associated with developing viral disease states across multiple viral infections in salmon, with analyses including six acute and chronic viral diseases in salmon. Validation studies applied gene paralog-specific qRT-PCR assays across salmon species from multiple IHNv challenge studies and from a natural outbreak of a suspected viral-induced jaundice syndrome disease in farmed Chinook salmon. Most of the candidate VDD biomarkers showed discrimination between latent and disease states across experimental study sets. Only 6 of the 45 unique biomarkers (CA038063, ZFP9, GNL3, MHCIuba, PSMB8 and TRIM1) were not highly discriminating. Importantly, the VDD panel was differentially activated across tissues and could classify fish based both on host response in the primary infective tissue, but also in secondary tissues, including non-destructively sampled gill tissue; these data suggest that the chosen biomarkers were predictive of the development of a systemic viral disease state. Unsupervised analysis with feature selection based on iterative PCA as implemented in the gene shaving algorithm revealed that in all validation studies, a VDD panel with as few as nine biomarkers was capable of separating viral disease from latent infections and bacterial diseases. The VDD panel applied to non-destructive gill biopsies from wild-migrating salmon smolts showed strong clustering of fish that was highly correlated with IHNv detections. However, as we showed in the challenge validation studies, there was not always a one-to-one correspondence with IHNv detection in gill in the waterborne study (the more natural of the two). Gill is a primary route of entry of the virus, and we know from the waterborne challenge studies that the virus can be detected in gill soon after exposure and prior to inducing damage associated with disease development in other tissues. Moreover, given the very small gill biopsies taken (tips of 1–2 gill filaments), we suspect that some samples tested were false IHNv-negative. This is an important demonstration, as it shows that the mere presence of the virus in any tissue does not necessarily indicate presence of disease. Importantly, it also shows that molecular disease diagnostics may be possible using miniscule gill biopsy samples that cause little harm to the organism (Jeffries *et al.*, 2014) and could thus be a powerful tool to assess disease physiology in species of conservation concern.

In each of the validation studies listed above, including the IHNv challenges, there were often infectious agents other than the targeted virus (IHNv or PRv) present among the sampled fish. Indeed, the IHNv challenged salmon that tested negative for IHNv and were assumed disease/infection free pre-challenge carried a range of other infectious agents, both bacterial and parasitic, but generally only at background levels (low loads). Chinook salmon from the natural jaundice outbreak carried an even greater range of infectious agents, some present at appreciable loads. We demonstrated in each of these studies that not only did the presence of background infections not impact the resolution of a viral disease state, we found that only a few of the VDD biomarkers were weakly correlated (almost always negatively) with these non-viral agents (data not shown). Our final validation dataset, the aquaculture regulatory audit samples, provided the most difficult test for the robustness of the VDD biomarkers, including application on mixed tissue RNA (after already demonstrating that the VDD worked across tissues) from recently dead fish (RNA potentially partially degraded) that had been diagnosed with a large range of diseases (most not viral), most carrying a range of mixed infections (viral, bacterial, fungal and protozoan). These samples exemplified typical diagnostic samples for cultured fish. While salmon specifically diagnosed with characterized viral diseases (HSMI in Atlantic salmon and jaundice in Chinook salmon, both associated with PRv) were well discriminated from fish with the bacterial diseases rickettsiosis, vibriosis, and mouth rot, and disease caused by the microsporidian *Loma* parasite, BKD presented more difficulties, with many of the VDD biomarkers showing similar patterns of up-regulation, especially in Atlantic salmon. Interestingly, unlike most bacteria, the causative agent of BKD, *R. salmoninarum*, can survive and replicate intracellularly, subverting typically cellular defenses and instead eliciting an IFN-gamma response (Rhodes *et al.*, 2009) somewhat similar to viruses. However, we were able to identify a robust set of twelve VDD biomarkers that differentiated viral diseases from bacterial and parasitic diseases in both species most, if not all, of the time and were positively associated with PRv loads. These included PXMP2, IFIT5, GAL3, NFX1type, VIG4, DDX58, MX1, RSAD2 (aka viperin), VAR1, IFI44 and HERC6. Additional powerful biomarkers were identified for Pacific (Trim21, IFI) and Atlantic (CD9, RAD1, SACS, XAF1) salmon whereby assays did not work across species; if re-designed, these biomarkers may also contribute to the universal separation of viral disease states across species.

The final robust, universal VDD panel containing 11 biomarkers can classify salmon experiencing diseases caused by RNA viruses, but did not discriminate audit salmon carrying ENv, the causative agent of viral erythrocytic necrosis (VEN), previously EIBS (literature reviewed in Plumb, 1993). However, the histopathological diagnostics applied to these fish did not include blood smears necessary to resolve viral inclusion bodies characteristic of VEN; hence, the fish were not actually diagnosed with viral disease, so we cannot be sure that the VDD would not identify viral disease states for DNA viruses.

In humans, host transcription biomarkers have been developed for clinical application for viral influenza (Zaas *et al.*, 2009; Andres-Terre *et al.*, 2015), acute viral respiratory infections (Zaas *et al.*, 2009; Andres-Terre *et al.*, 2015), hepatitis C (Chen *et al.*, 2005) and tuberculosis (Lu *et al.*, 2011). These are all diseases whereby the infective agents are carried by a large portion of the population, with disease ensuing in only a fraction of those exposed; hence, the need for a means to distinguish between latent carriers and developing or active disease, and the potential to diagnose and target proactive therapeutants to asymptomatic patients essential. The two independent studies on acute viral respiratory diseases, including influenza, identified highly similar panels of genes capable of classifying viral disease prior to the onset of symptoms. These panels also showed considerable overlap with our VDD panel in salmon. The range of RNA viruses used to identify the salmon VDD was much broader than in the human studies, including Rhabdoviridae (IHNv and ISAv), Orthoreoviridae (PRv), Totiviridae (PMCv) and Birnaviridae (IPNv). ISAv is in the orthomyxovirus family containing influenza viruses. DDX58, HERC6, IFH1, IFIT5, IFI44, IRF7, GAL3, MX1, RSAD2, STAT1 and XAF1 were among the 30 biomarkers identified by Zaas *et al.* (2009) and/or 16 biomarkers from Andres-Terre *et al.* (2015) also significant in our study. In fact, RSAD2, a potential antiviral molecule (Chin and Cresswell, 2001), was the most highly differentially expressed gene in the Zaas *et al.* (2009) study. Other gene families were also highly overlapping between the human and salmon studies, including interferon-induced proteins (IFI salmon versus IFI1, IFI27), interferon-induced proteins with tetratricopeptide repeats (IFIT5 salmon versus IFIT1, IFIT2, IFIT6), receptor transporter proteins (RTP3 salmon versus RTP4) and E3 ubiquitin-protein ligases (HERC6 versus HERC5/HERC6). RSAD2 and MX1 were also among the 18 biomarkers differentiating responders and non-responders for hepatitis C treatment (Chen *et al.*, 2005). In the Andres-Terre study, functional analysis revealed the key transcriptional regulators of their 11 robust influenza biomarkers were IFR7 and STAT1; our study resolved these genes among those in the VDD, although not among our top 11. IFNG was the most significant transcriptional regulator of our VDD biomarker panel, with 16 of the 41 annotated VDD under IFNG regulation. A total of 18 genes were associated with viral reproduction, 16 with viral diseases, 12 with viral response, 7 with viremia, 6 with viral clearance and 4 with response to dsRNA. Protein ubiquination, innate and adaptive immune response, inflammation and infectivity were also over-represented biological processes. Network analysis in Pathway Studio^TM^ also revealed a plausible gene network involved in host viral response.

The salmon viruses used in the development of VDD biomarkers are known or suspected to cause disease in cultured salmon, but for some, we know little of their effects on wild salmon. IHNv is the exception, as this virus is endemic to Sockeye salmon and Rainbow trout populations on the West coast of North America and can cause considerable losses of juvenile (fry-smolt) salmon in freshwater (Wolf, 1988); this virus has also spread to Europe and Asia (Dixon *et al.*, 2016). The level of IHNv impact is highly species-specific. Sockeye salmon are the most susceptible Pacific salmon species, especially at the egg to fry stage, with populations from British Columbia to Alaska suffering epizootics (Williams and Amend, 1976; Follet and Burton, 1995). Chum and Chinook salmon exposed to IHNv in the laboratory can develop disease, and we demonstrated that the VDD identified the development of disease in one of these species, but direct effects on survival were low in our challenge studies. What is not known, however, is whether indirect effects of IHN, e.g. impacts on physiological performance, may contribute to reduced survival, although impacts on swim performance have been demonstrated (LaPatra *et al.*, 1995), and recent research by our group indicates that IHNv-infected salmon are at increased risk of predation (Furey, 2016).

Other than IHN and VEN (caused by a DNA iridovirus), there have been few reports of viral disease in wild salmon, despite their known sometimes devastating impacts on cultured fish (Bakke and Harris, 1998). ISAv is perhaps the second most studied virus in a wild context. ISAv causes acute fatal systemic infections in marine-farmed Atlantic salmon (Kibenge *et al.*, 2004), with epidemics reported in Chile (Mardones *et al.*, 2014), Norway (Lyngstad *et al.*, 2008), Scotland (Murray *et al.*, 2010) and the eastern coast of North America (Gustafson *et al.*, 2007). Wild sea running Brown trout are the proposed marine reservoir for avirulent wild-type ISAv in Norway, yet there have not been any documented cases of the disease ISA in Brown trout (Nylund *et al.*, 1995; Plarre *et al.*, 2005). Moreover, while most salmonid species can become infected by ISAv, the virus is only known to cause disease and mortality in Atlantic salmon, and the only documented cases of ISA are in farmed Atlantics (reviewed in Plarre *et al.*, 2005). ISAv virulence derives from deletions in the stalk region of the HE protein and insertions near the proteolytic-cleavage site of the precursor F0 protein (Plarre *et al.*, 2012). Wild-type ISAv found naturally in Brown trout populations does not contain the deletion in the stalk region in segment 6; it is hypothesized that this deletion may predominantly occur in Atlantic salmon under high density culture (Nylund *et al.*, 2003). When it does occur in wild salmon, it could impose unobserved mortality in smolts in the early marine environment (Uno, 1990), but is expected to be highly limited in transmission potential due to high pathogenicity (Bakke and Harris, 1998).

Viruses PMCv, PRv and Salmon alphavirus (SAv), and cause chronic, slow progressing diseases in farmed Atlantic salmon, with (generally low) mortality occurring over several months (McLoughlin *et al.*, 2002; Poppe and Sejerstad, 2003; Kongtorp *et al.*, 2004). Given their chronic nature, these infectious agents can be present and shed from salmon within farmed populations for a prolonged period of time, which theoretically enhances their risk of transmission to and within wild fish populations. Importantly, the diseases caused by these viruses, all of which cause inflammation of the heart, can affect swimming behavior, causing either lethargy or erratic swimming (McLoughlin *et al.*, 2002; Kongtorp *et al.*, 2004; Haugland *et al.*, 2011), sub-lethal physiological impacts that may not be detrimental to farmed fish (i.e. a slow day on the farm) but carry significantly enhanced risk of predation in wild fish. However, while two of the viruses have been observed in wild salmon (PMCv and PRv) (Garseth *et al.*, 2012, 2013; Siah *et al.*, 2015) to date there is evidence of (mild) disease in wild populations only for PMCv (Poppe and Sejerstad, 2003). PRv is the only virus of the three detected in the Pacific Northwest (Miller, unpublished data), being fairly ubiquitous in farmed Atlantic and Chinook salmon, and detected in most Pacific salmon species (Marty *et al.*, 2015), albeit at considerably lower prevalence. PRv has been associated both with HSMI in Atlantic salmon and jaundice syndrome-related diseases in Pacific salmon in Norway (Rainbow trout—Olsen *et al.*, 2015) and Chile (Coho salmon—Godoy *et al.*, 2016). While challenge studies with the North American strain of PRv (98% similar to strains in Norway) have not resulted in compelling evidence of disease (Garver *et al.*, 2015, 2016), clearly both diseases described in farmed salmon in Norway and Chile do exist in association with PRv in BC (Di Cicco *et al.*, 2017; Miller, unpublished data), and wild fish with the outward appearance of jaundice (yellowing of the belly and under the eye) have been observed. The fact that this virus can be observed in both farm and wild settings, sometimes at modest to high loads, in the absence of histological presentation of disease, has caused some to question whether PRv can cause disease in wild fish (Garseth *et al.*, 2013; Marty *et al.*, 2015). However, our analyses of farm audit salmon provided evidence that the VDD biomarkers were able to discriminate fish diagnosed with HSMI (Atlantic salmon) and jaundice (Pacific salmon), both associated with PRv, from viral negative fish and from fish diagnosed with bacterial or parasitic diseases.

For many viruses, challenge studies have already demonstrated impacts on physiological performance, which as suggested previously, may enhance impacts of sub-lethal disease in wild fish. Secondary impacts associated with enhanced predation risk may ensue if visual acuity, swim performance, and/or feeding and growth are affected (Miller *et al.*, 2014). Impacts on swim performance have been demonstrated in association with disease from IHNv, ISAv, IPNv, VHSv (Meyers, 2006), PMCv (Haugland *et al.*, 2011) and PRv (Kongtorp *et al.*, 2004). Impacts on feeding and growth, which may also have ramification on size-selective predation and energetic potential for predation escapement, have also been demonstrated for IPNv (Meyers, 2006), PRv (Kongtorp *et al.*, 2004), SAv (McLoughlin *et al.*, 1998) and VHSv (Baulaurier *et al.*, 2012). Enhanced pathogenicity has been demonstrated for several viruses in association with elevated water temperatures (IHNv—La Patra *et al.*, 1979, IPNv—Dobos and Roberts, 1983, VEN/ENV—Korsnes *et al.*, 2005). As a result, these viruses may show stronger impacts on both wild and farmed salmon in a warming climate.

While tools merging disease-predictive host biomarkers with broad-based pathogen monitoring could be of high relevance to human and veterinary health diagnostics fields, they are equally important for disease studies in natural systems whereby sick and dying individuals are not readily available for diagnosis. The identification of a unique set of biomarkers that can differentiate across viral species latent infections from disease states underscores the conserved nature of the host response to viral infection that even crosses broad species borders (humans to fish). Given the consistency across salmon and human studies, it is highly probable that many of these biomarkers will be transportable to viral diseases in other wildlife species. Hence, this molecular diagnostic technology could begin to fill the need for better diagnostics capabilities to identify a wide range of pathogens and infectious diseases in wildlife (Deem *et al.*, 2001). Molecular diagnostics applied in conjunction with biotelemetry studies can further demonstrate whether there is an association between migratory survival and infection and/or disease status of individuals. If combined with predation studies, as in Furey (2016) or Miller *et al.* (2014), one could also demonstrate whether diseased individuals are more susceptible to predation, and given knowledge of the pathogens present, which diseases are likely associated with greater risk. All of this information can be gained with a conservation-based approach that does not require lethal sampling to demonstrate disease (as is required with traditional histopathology), and can be effectively applied even in situations where individuals with late-stage diseases are rare.

## Supplementary Material

Supplementary Data
